# Bifunctional octadentate pseudopeptides as Zirconium-89 chelators for immuno-PET applications

**DOI:** 10.1186/s41181-024-00263-1

**Published:** 2024-05-06

**Authors:** Valentina Albanese, Chiara Roccatello, Salvatore Pacifico, Remo Guerrini, Delia Preti, Silvia Gentili, Matteo Tegoni, Maurizio Remelli, Denise Bellotti, Jonathan Amico, Giancarlo Gorgoni, Emiliano Cazzola

**Affiliations:** 1 Department of Environmental and Prevention Sciences, University of Ferrara, Palazzo Turchi di Bagno, C.So Ercole I d’Este 32, 44121 Ferrara, Italy; 2 Department of Chemical, Pharmaceutical and Agricultural Sciences, University of Ferrara, Via Luigi Borsari 46, 44121 Ferrara, Italy; 3 Department of Chemistry, Life Sciences and Environmental Sustainability, University of Parma, Parco Area Delle Scienze 11/A, 43124 Parma, Italy; 4 Department of Radiopharmaceutical, IRCCS Sacro Cuore Don Calabria Hospital, Via Don A. Sempreboni 5, 37024 Negrar di Valpolicella, Verona, Italy

**Keywords:** Pseudopeptide chelator, Zirconium-89 (^89^Zr), Molecular imaging, Immuno-PET, Clinical oncology

## Abstract

**Background:**

Positron emission tomography (PET) is a highly sensitive method that provides fine resolution images, useful in the field of clinical diagnostics. In this context, Zirconium-89 (^89^Zr)-based imaging agents have represented a great challenge in molecular imaging with immuno-PET, which employs antibodies (mAbs) as biological vectors. Indeed, immuno-PET requires radionuclides that can be attached to the mAb to provide stable in vivo conjugates, and for this purpose, the radioactive element should have a decay half-life compatible with the time needed for the biodistribution of the immunoglobulin. In this regard, ^89^Zr is an ideal radioisotope for immuno-PET because its half-life perfectly matches the in vivo pharmacokinetics of mAbs.

**Results:**

The main objective of this work was the design and synthesis of a series of bifunctional octadentate pseudopeptides able to generate stable ^89^Zr complexes. To achieve this, here we investigated hydroxamate, *N*-methylhydroxamate and catecholate chelating moieties in complexing radioactive zirconium. *N*-methylhydroxamate proved to be the most effective ^89^Zr-chelating group. Furthermore, the increased flexibility and hydrophilicity obtained by using polyoxyethylene groups spacing the hydroxamate units led to chelators capable of rapidly forming (15 min) stable and water-soluble complexes with ^89^Zr under mild reaction conditions (aqueous environment, room temperature, and physiological pH) that are mandatory for complexation reactions involving biomolecules. Additionally, we report challenge experiments with the competitor ligand EDTA and metal ions such as Fe^3+^, Zn^2+^ and Cu^2+^. In all examined conditions, the chelators demonstrated stability against transmetallation. Finally, a maleimide moiety was introduced to apply one of the most promising ligands in bioconjugation reactions through Thiol-Michael chemistry.

**Conclusion:**

Combining solid phase and solution synthesis techniques, we identified novel ^89^Zr-chelating molecules with a peptide scaffold. The adopted chemical design allowed modulation of molecular flexibility, hydrophilicity, as well as the decoration with different zirconium chelating groups. Best results in terms of ^89^Zr-chelating properties were achieved with the N-methyl hydroxamate moiety. The Zirconium complexes obtained with the most effective compounds were water-soluble, stable to transmetallation, and resistant to peptidases for at least 6 days. Further studies are needed to assess the potential of this novel class of molecules as Zirconium-chelating agents for in vivo applications.

**Supplementary Information:**

The online version contains supplementary material available at 10.1186/s41181-024-00263-1.

## Background

Positron emission tomography (PET) is a diagnostic imaging technique broadly used for the characterization, treatment planning and therapy monitoring of diseases, most notably cancer (Gambhir 2002; Zeglis and Lewis 2011). Immuno-PET is an emerging application of PET that combines this methodology with the high specificity and affinity of monoclonal antibodies (mAbs) in cancer targeting, to generate tomographic information (Holland 2018; Wei and Rosenkrans 2020; Yoon and Park 2020; Sharma and Glaser 2021). Specifically, immuno-PET allows to monitor tumor response measuring cancer-associated antigens, offering the possibility to customize therapy and quantitatively measure the in vivo distribution of mAbs (Wei and Rosenkrans 2020).

Zirconium-89 (^89^Zr) has been extensively investigated as a useful radionuclide to employ as immuno-PET imaging tool. The half-life of ^89^Zr (3.27 days) nicely fits with the in vivo bio-distribution and pharmacokinetic properties of radioactive antibodies allowing the selective delivery of radiation on biological targets overexpressing specific cancer biomarkers (Liu 2008).

**Fig. 1:**
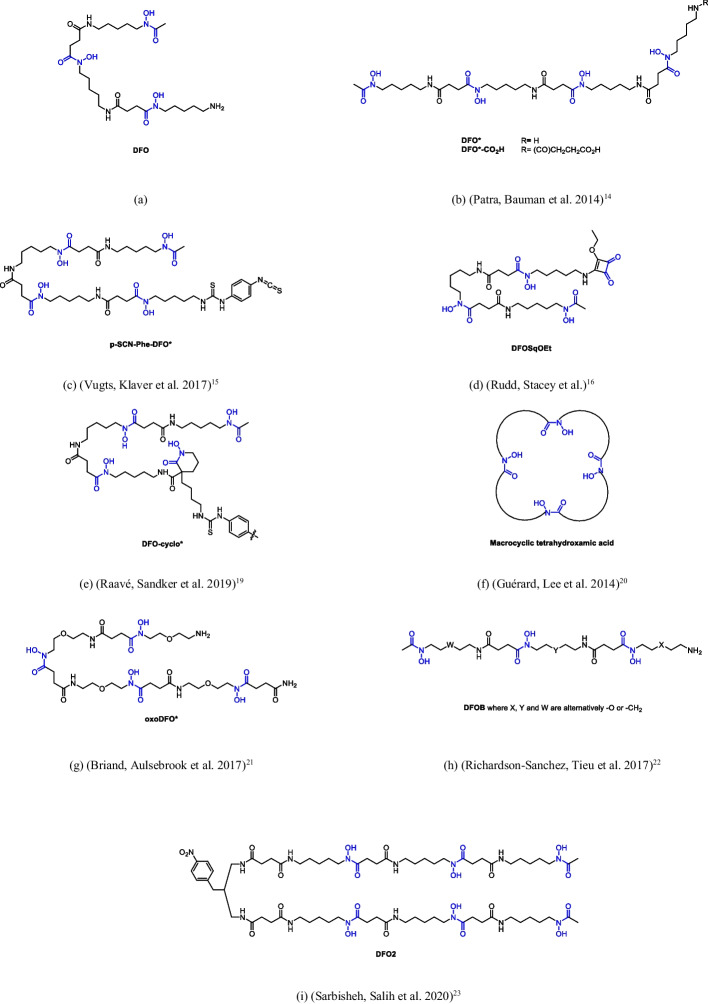

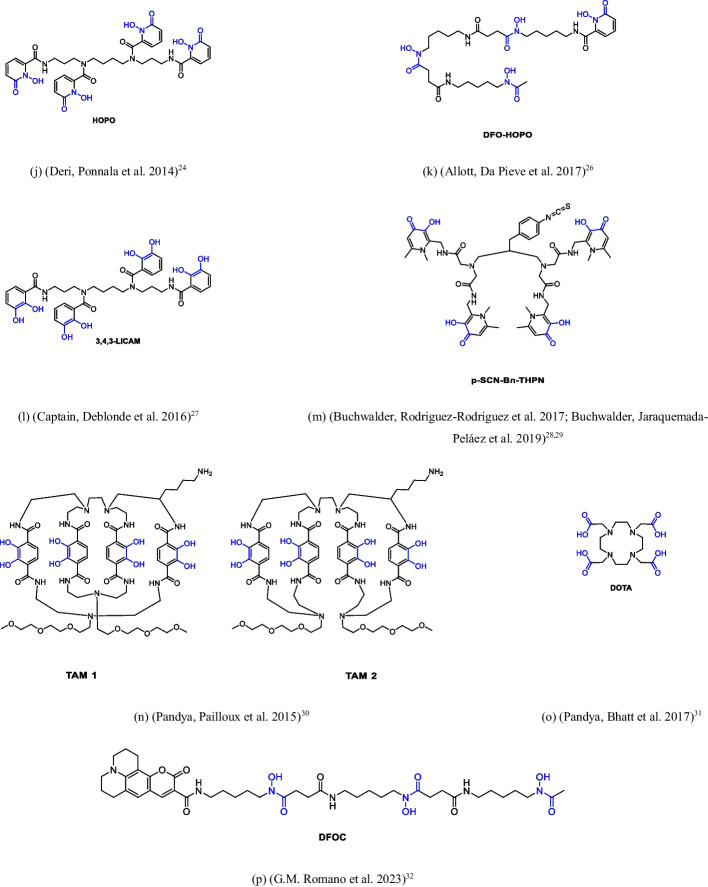
DFO and tetra- and exa-functional chelators reported in the literature as ^89^Zr ligands

To date, Desferrioxamine (DFO, Fig. 1a), a natural siderophore expressed by the bacteria *Streptomyces pilosus* and clinically developed as Fe^3+^ chelator for the treatment of iron overload in patients with β-thalassemia (Crisponi and Remelli 2008; Bellotti and Remelli 2021) is the most investigated chelating agent for ^89^Zr (Dilworth and Pascu 2018). Holland et al. (2010, 2020) demonstrated that [^89^Zr]Zr-DFO is an octadentate complex involving the six-binding oxygens of DFO hydroxamate moieties and two additional water molecules that contribute to the completion of the ^89^Zr coordination sphere. The two water molecules are probably responsible for the limited in vivo stability of the [^89^Zr]Zr-DFO complex, encouraging the development of more stable radiotracers for human applications. In fact, high stability of ^89^Zr-based radiotracers is crucial to prevent the accumulation of the radionuclide in bone to ensure high-resolution PET images (Pandya and Henry 2020). Moreover, for in vivo application, it has to be considered that the radiotracer is generally injected in the bloodstream at very low concentrations, exposing it to endogenous competitive cations and natural moieties with chelating properties that may challenge the stability of the metal chelate. This evidence underlines the clinical relevance of developing novel chelators that can generate stable octacoordinated species with ^89^Zr, avoiding its release during the circulation time after injection.

In the past few years, several efforts have been devoted to the design and development of [^89^Zr]Zr-DFO complexes with improved properties. Patra et al. (2014) hypothesized that a chelator bearing four hydroxamate moieties (DFO*, Fig. 1b) could provide an octadentate-complex with ^89^Zr, resulting in a ligand with a better profile for application in PET methodologies. In the same paper, the synthesis of the succinic acid derivative of DFO* (DFO*–COOH, Fig. 1b), was also reported: the latter is a bifunctional chelating agent (BFCA) containing a terminal carboxyl group available for conjugation with a bioactive molecule through the formation of a peptide bond. The conjugation of DFO*-COOH (extended at its N-terminus with a βAla_3_ spacer) with a modified minimum binding sequence of the peptide bombesin (BBS), [Nle^14^]BBS(7–14) (QWAVGHLNle-NH_2_), was also described. On the basis of transchelation challenging experiments, the [^89^Zr]Zr-DFO*(βAla)_3_[Nle^14^]BBS(7–14) radioconjugate proved much more stable than the corresponding DFO analogue. Few years later, the same research group reported both the synthesis of the bifunctional ligand *p*-SCN-Phe-DFO* (prepared from DFO* and *p*-phenylenediisothiocyanate, Fig. 1c) and its conjugation with the monoclonal antibody trastuzumab; the chelator was then radiolabelled with ^89^Zr (Vugts and Klaver 2017). Both in vitro and in vivo stability of ^89^Zr-DFO*-trastuzumab were higher than those of ^89^Zr-DFO-trastuzumab. A potentially octadentate new chelator for Zr(IV) was described by Rudd and coworkers (Rudd and Roselt 2016); they reacted DFO mesylate with diethyl squarate in a basic environment, obtaining, with an excellent yield, the corresponding squaramide ester (DFOSqOEt, Fig. 1d). The synthesis of this DFO derivative is particularly simple because it does not require the protection of the hydroxamic groups. The second squarate ester is then able to quickly react with the amino groups of proteins or antibodies, to give a further stable vinyl amide bonding. Notably, the squarate group also contains a dione, whose donor oxygens can complete the coordination sphere of zirconium, thus enhancing the stability of the chelate. In addition, the squaramide ester derivatives have excellent shelf stability and water solubility. DFOSqOEt was initially conjugated with trastuzumab, in order to study its potential as an antibody marker and its possible PET applications (Rudd and Roselt 2016). Very recently, an automated procedure was described for the clinical production of the conjugate [89Zr]Zr-DFOSq-Durvalumab (Wichmann et al. 2023) and a short report, describing an ImmunoPET Phase 0 study on 5 volunteers suffering from lung cancer was published (Hegi-Johnson et al. 2023). The aims this study were both to assess the safety of the conjugate chelator, to determine its biodistribution and to define the optimal conditions for subsequent clinical studies.

Similarly, in vitro and in vivo stabilities of ^89^Zr-labelled DFOcyclo* (Fig. 1e) and DFOcyclo*-trastuzumab were compared to those of DFO and DFO-trastuzumab by Raavé et al. (2019) DFOcyclo* is an octadentate derivative of DFO, obtained by adding a cyclic hydroxamic group. The in vitro stability of radiolabelled DFOcyclo* was higher than DFO and comparable to DFO*, upon the addition of an excess of EDTA. Among the three conjugated with trastuzumab, the one based on DFO was less stable in vitro than those containing DFO* or DFOcyclo*. The radiolabelled DFOcyclo*, conjugated with trastuzumab using isothiocyanate as a linker, showed an excellent in vivo stability in tumour-bearing nude mice.

Three tetra hydroxamate macrocyclic DFO derivatives have also been investigated, characterized by a variable ring (and cavity) size (Fig. 1e) (Guérard, et al. 2014). The rings contained 28, 32 and 36 atoms, respectively, with 5-, 6-, or 7-carbon alkyl chains separating the hydroxamic functional groups. The complex stability and inertness proved to increase with the size of the cavity, becoming better with respect to DFO complexes. This effect was attributed not only to the octadentate capability of the new ligand but also to the suitability of the cavity size, the rigidity of the complex, and the macrocyclic effect.

Despite the promising chemical features of Zr complexes with both linear and cyclic DFO derivatives, the low solubility in water prevented their further development as radiotracer for in vivo experiments. The solubility of Zr-chelating agents was improved by means of the insertion of hydrophilic ether groups in the chain: the synthesis of oxoDFO* (Fig. 1f) and its isothiocyanate BFCA has been realized through a simple solid phase-assisted approach (Briand and Aulsebrook 2017). The synthesis of a hydrophilic tetrahydroxamate chelator was also independently realized by Codd and coworkers, using a “microbiological-chemical synthetic” approach (DFOB-O_3_–PBH-O_1_, Fig. 1g) (Richardson-Sanchez and Tieu 2017), and its complexation ability towards ^nat^Zr(IV) ion was confirmed by HLPC-MS, after an incubation time of 15 min at neutral pH, in aqueous solution and room temperature.

A new linear and high-denticity chelator, derived from DFO and containing six hydroxamic groups with 12 available donor atoms (DFO2, Fig. 1h) has been very recently described (Sarbisheh and Salih 2020). The modular synthetic scheme required two steps: (i) reaction of DFO mesylate with succinic anhydride to obtain the DFO-COOH derivative and (ii) its dimerization by reaction with a propylenediamine linker. The new chelator showed a very good coordination ability towards the Zr(IV) ion (much better that DFO), investigated trough DFT calculations, several in vitro stability assays and radiolabeling with ^89^Zr. Unfortunately, the solubility of DFO2 in water is poor, as expected.

Besides hydoxamate, other chemical groups have been reported in the literature and investigated as effective binding sites for Zr(IV). An octadentate ligand containing four hydroxypyridinone groups (HOPO, Fig. 1i) and its bifunctional derivative *p*-SCN-Bn-HOPO have been synthesized by Deri et al*.* ( 2014, 2015) Allot et al. (2017) added a hydroxypyridinone chain to DFO to form a new octadentate ligand with a mixed chelating mode (DFO-HOPO, Fig. 1j). The catechol moiety was instead chosen to synthesize 3,4,3-LI(CAM) (Fig. 1k) (Captain and Deblonde 2016). A tetrapodal 3-hydroxy-4-pyridinone (THPN) and its bifunctional derivative p-SCN-Bn-THPN (Fig. 1l) have also been recently investigated (Buchwalder and Rodriguez-Rodriguez 2017; Buchwalder and Jaraquemada-Peláez 2019).Moreover, two di-macrocyclic terephthalamide ligands (BFC1 and BFC2, Fig. 1m) have been described (Pandya and Pailloux 2015). Finally, several polyazamacrocyclic ligands have been synthesized and tested by Pandya et al. (2020, 2017) as mixed nitrogen/oxygen Zr(IV) chelators, able to very effectively saturate the metal coordination positions; among them, DOTA (Fig. 1n) is especially interesting since it is a widely used (and characterized) ligand (especially for gadolinium) in diagnostic imaging. Unfortunately, these macrocyclic ligands generally suffer from low solubility. A new DFO derivative (DFOC, Fig. 1o) has been very recently synthesized by the conjugation of DFO with coumarin, in order to impart fluorescence properties. Metal chelation properties of DFOC proved very similar to those of natural DFO, but the presence of the fluorescent coumarin moiety makes DFOC suitable as dual imaging (PET/fluorescence) ^89^Zr probe (Romano et al. 2023).

**Fig. 2:**
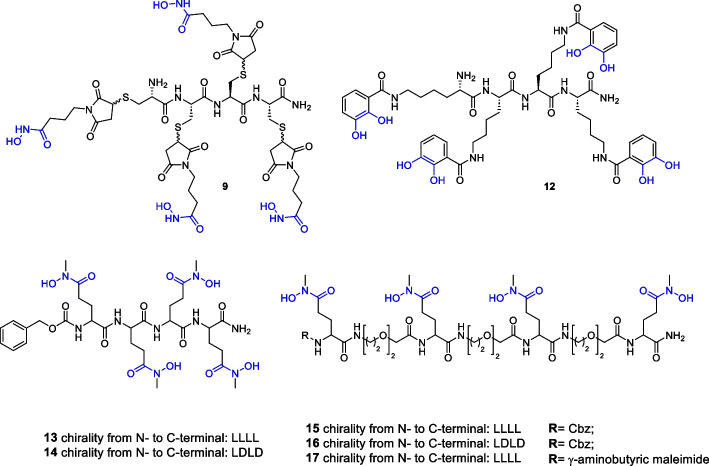
Novel ^89^Zr ligands synthesised in this work

Based on the above literature survey, we designed and synthesized some new peptide-based ligands, containing different chemical moieties able to generate stable octacoordinated species with ^89^Zr (see compounds **9** and **12**–**17**, Fig. 2). The well-established methodology for the solid phase as well as in solution synthesis of the peptide backbone associated with the availability of amino acids characterized by side chain with different chemical moieties were conveniently applied for generating novel ^89^Zr-chelating molecules. In our chemical design the peptide backbone has been modulated in terms of chirality and hydrophilicity and decorated with hydroxamate as well as catechol moieties as Zr-chelating units. Finally, a maleimide function has been introduced to allow the further reaction with antibodies. The novel molecules have been evaluated for their ability to chelate Zr in mild conditions and to provide stable and water soluble chelates.

## Results

### Synthesis

**Scheme 1:**
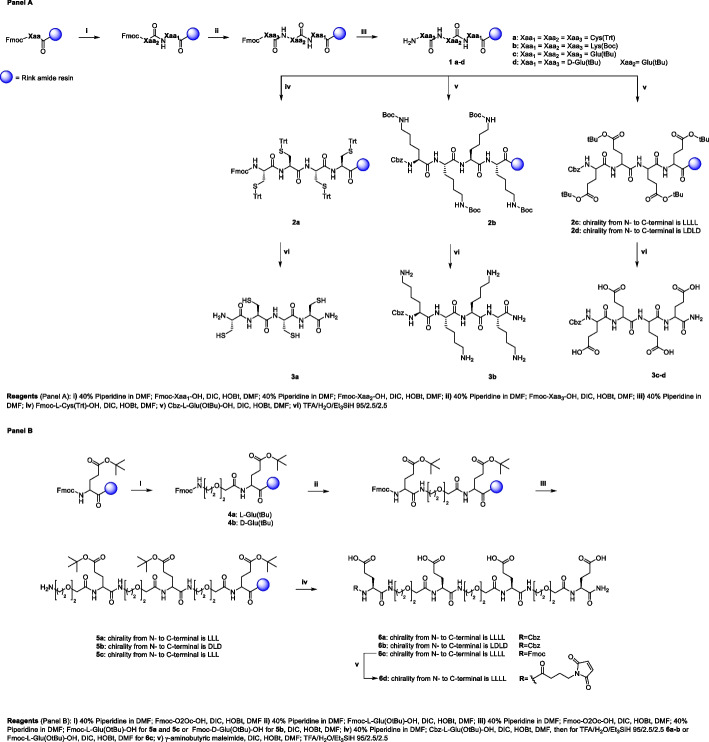
Solid phase peptide synthesis of intermediates **3a**-**d** and **6a**-**d**

The synthetic approach leading to our target pseudopeptide chelators started from the solid phase peptide synthesis (SPPS) of **3a–d**, as depicted in Scheme 1 (Panel A). Compounds **1a–d** were synthesised on Rink amide solid support through a DIC/HOBt coupling strategy, in which three protected Cys, Lys or Glu residues, were sequentially coupled, respectively. The coupling between a Fmoc-Cys(Trt)-OH residue and derivative **1a** gave the tetracysteine scaffold **2a**. Fmoc deprotection of **2a** was required before the cleavage of the tetracysteine **3a** from the solid support and the simultaneous removal of the S-trityl protecting groups. Peptide scaffolds **3b**, **3c** and **3d** were obtained by adding a Cbz-Lys(Boc)-OH or a Cbz-Glu(tBu)-OH residue to the precursors (**2b–d**), respectively, prior to classical TFA-mediated cleavage.

The N-terminal protected pseudopeptides **6a**-**b** were obtained through a similar SPPS approach (Scheme 1, panel B) based on alternative couplings with O2Oc and L- or D-Glutamate residues. This allowed the introduction of oxyethylene units between the four glutamic residues of **3c**-**d**. In the case of **6c**, a final condensation with γ-aminobutyric maleimide was performed to give **6d**.

For the synthesis of **9**, an O-protected hydroxamate precursor was designed in order to avoid potential side-reactions of a primary hydroxamate in the multi-step synthetic approach. Thus, the maleimide derivative** 7** was obtained via a coupling reaction between γ-aminobutyric maleimide and O-tert-Butyl- hydroxylamine (Lambert and Danishefsky 2006; Song and Ngai 2009; Sinclair et al. 2009). Compound **3a** was treated with **7** via a thiol-Michael reaction providing **9** after O-tertbutyl deprotection of **8**.

The synthesis of the tetra-lysine derivative **12** was performed in good yield through a first HATU-mediated coupling reaction between **3b** and the protected cathecolate (Captain and Deblonde 2016) **10** providing the linear tetralysine-catecholate **11**. The final acidic treatment allowed the removal of both N-terminal Cbz and catechol diphenyl methylene acetal protecting groups.

**Scheme 2:**
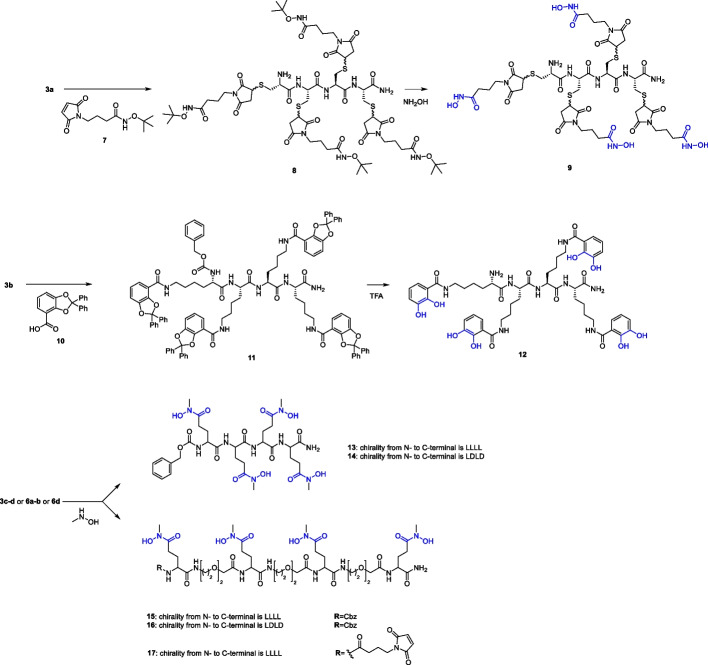
Synthetic approach for the introduction of chelating moieties on pseudopeptide scaffolds **8**, **11**, **3c**-**d**, **6a**-**b** and **6d**

The target Zr chelators **13**–**17** (Scheme 2) were obtained through combined solution/solid phase strategy. Compounds **3c**-**d**,** 6a**-**b** and **6d** were reacted with *N*-methylhydroxylamine in the presence of HATU and DIPEA to give **13**–**14** and **15**–**17**, respectively.

### Evaluation of Zirconium-89 complexation and stability studies

The kinetics of complex-formation and stability over time have been evaluated using different chelation protocols (see “Materials and methods: Complexation protocols” section) to identify the ideal reaction conditions with the radioactive metal. The chelators were evaluated for radiolabeling efficiency with both [^89^Zr]Zr-chloride ([^89^Zr]ZrCl_4_) and [^89^Zr]Zr-oxalate ([^89^Zr]ZrOx_2_) formulations as precursors of the radioactive isotope. Radiometal precursor solutions were prepared on site, following standard protocols (Cisternino and Cazzola 2021 Nov; Perk et al. 2010).

With the aim to identify potential peptide-based Zirconium-89 chelators we firstly considered the best chelating functions suggested from literature (Tinianow and Gil 2010; Ma and Meszaros 2015). Among these, hydroxamate and catechol groups were reported as the most promising Zirconium-89 bidentate chelating units and an additional advantage of these functional groups is their ease of introduction in peptide sequences. In an early stage of our work, we investigated the feasibility of synthesizing the catecholate-based tetrapeptide ligand **12** and we also focused our attention on the two linear peptide derivatives **9** and **13**: the first one bearing primary hydroxamic functions and the other one with *N*-methyl-substituted hydroxamic units. We performed an initial screening of these new chelating agents (**9**, **12** and **13**), which made it possible to assess the kinetics of complex formation with ^89^Zr and the stability of these chelates over time. The radiolabeling efficiency was determined by radio-iTLC analyses using EDTA 50 mM as mobile phase (Table 1). The experiments were accomplished in different conditions using various temperatures and pH to determine the optimal Zirconium-89 radiolabelling protocols. As a first step, we intended to determine the reactivity of the new peptide ligands and, to this purpose, we employed [^89^Zr]ZrCl_4_ that was also reacted with DFO-Bz-NCS, as reference chelator for Zirconium-89 (Sciacca et al. 2021).

**Table 1:** Radiolabeling efficiency of [^89^Zr]Zr-DFO-Bz-NCS, [^89^Zr]Zr-Compd **9**, [^89^Zr]Zr-Compd **12** and [^89^Zr]Zr-Compd **13**

Time											
	[^89^Zr]Zr-DFO-Bz-NCS		[^89^Zr]Zr-Compd 9			[^89^Zr]Zr-Compd 12			^89^Zr]Zr-Compd 13		
	RT	50 °C	RT	50 °C	90 °C	RT	50 °C	90 °C	RT	50 °C	90 °C
*pH 3.5*											
15 min	100	100	15	5	0	100	82	100	20	52	22
30 min	100	100	0	0	10	100	100	100	33	100	16
60 min	100	100	0	0	25	100	100	100	100	100	23
*pH 7.0*											
15 min	80	79	18	30	25	24	44	21	25	52	17
30 min	100	100	23	22	27	37	32	10	31	26	10
60 min	100	100	29	0	25	32	18	12	39	37	10

Radiolabeling efficiency measured in duplicate using iTLC eluted with a mobile phase consisting of 50 mM EDTA, at room temperature, 50 and 90 °C. The percentage of complex-formation is obtained by evaluating the intensity of the radioactive spots corresponding to the complex and to the free zirconium

The kinetics of complex-formation estimated for compounds **9**, **12** and **13** from the radio-iTLC technique using [^89^Zr]ZrOx_2_ instead of [^89^Zr]ZrCl_4_ were generally comparable (data reported in Additional file 1: Table S1 of the Supporting Infomation). In both cases, it has been observed that the complex [^89^Zr]Zr-DFO-Bz-NCS was formed more rapidly than in the case of our ligands with 100% formation after only 15 min at room temperature with a quantitative radiochemical yield. Nevertheless, the protocols starting from [^89^Zr]ZrCl_4_ showed an excellent response for the ligands **12** and **13** under acidic conditions at room temperature. In fact, within 60 min, the reactions were quantitative, and, under the same conditions, the complexes were stable for more than 6 days, demonstrating good reactivity and stability over time (see Additional file 1: Fig. S1). The chelator **9** appeared to be the least promising Zirconium-89 chelator as it only reached a 30% of complex formation at 50 °C. As for the compounds **12** and **13**, interesting complexing behaviours were observed also with [^89^Zr]ZrOx_2_ at a pH of 7.0 at 37 °C. These results led us to further investigate their chelating ability using radio-HPLC analyses. The HPLC experiments with **13** confirmed the formation of the complex highlighting the presence of a single peak at a different retention time than that of [^89^Zr]ZrOx_2_. Same behaviour, with both zirconium-89 precursors, in terms of reactivity and stability, was show from **14** but with low reaction yield and less stability (see Additional file 1: Table S2). Unfortunately, it was not possible to further investigate the complex with the catecholate derivative **12** due to the formation of a precipitate in most organic solvents, data confirmed by the lack of radioactivity measured on the supernatant.

Starting from [^89^Zr]ZrOx_2_, the complex-formation kinetics of **15** and **16** with Zirconium-89 was also investigated. The results, reported in Table 2, confirm the ability of these molecules to quickly coordinate the metal and the stability tests showed an efficiency comparable to the chelator of choice, DFO-Bz-NCS.

**Table 2:** Radiolabeling efficiency of [^89^Zr]Zr-DFO- Bz-NCS, [^89^Zr]Zr-Compd **15** and [^89^Zr]Zr-Compd **16**

Time	Radiolabeling efficiency (%)					
	[^89^Zr]Zr-DFO-Bz-NCS		[^89^Zr]Zr-Compd 15		[^89^Zr]Zr-Compd 16	
	RT	50 °C	RT	50 °C	RT	50 °C
*pH 7.0*						
15 min	100	100	100	100	100	100
30 min	100	100	100	100	100	100
60 min	100	100	100	100	100	100

Radiolabeling efficiency measured in duplicate using iTLC eluted with a mobile phase consisting of 50 mM EDTA (pH 7.0, RT and 50 °C)

After assessing the complex formation kinetics with radioactive Zirconium-89 we carried out complexation experiments with the cold metal (^nat^Zr) for compounds **15** and **16**, in order to better clarify the complex-formation behaviour of these compounds (See “Complexation studies” section in SI).

### Challenge experiments and in vitro stability of [^89^Zr]Zr-15 and [^89^Zr]Zr-16

**Fig. 3:**
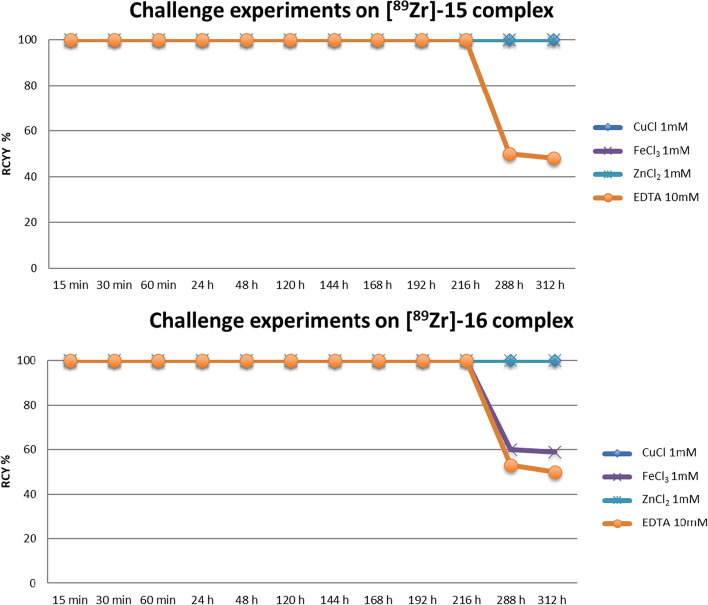
Challenge experiments conducted using [^89^Zr]ZrOx_2_. The graph shows the values resulting from the iTLC used to study the stability of the complexes between **15** and **16** with [^89^Zr]ZrOx_2_ in the presence of competing metals

Competition studies in which complexes are subjected to a challenging environment with an excess of biometals and/or biochelators can assess the ligand ability to give a stable chelate. In this kind of assays, the typical concentrations of potential competitors are much higher than that of the radiolabelled complex, thus requiring high ligand affinity for the radiometal. These tests are usually monitored via iTLC, HPLC and LC–MS techniques (Wadas and Wong 2010). Here we reported the results from the iTLC analyses (see “Materials and methods: Complexation protocols” section) which we performed for evaluating the inertness of complexes [^89^Zr]Zr-**15** and [^89^Zr]Zr-**16**. The ligands showed high solubility in water differently from recently reported octadentate ligands which seem to be sparingly soluble in aqueous media. (Dilworth and Pascu 2018; Richardson-Sanchez and Tieu 2017) We have chosen to investigate the stability of these complexes in different conditions for simulating situations that can occur in vivo or during their preparation. EDTA represents a particularly strong ligand challenge, since it forms very stable complexes with zirconium, although their kinetic instability in vivo precludes any use in medicine. [^89^Zr]Zr-**15** and [^89^Zr]Zr-**16** complexes were incubated with an excess of EDTA (ca. 120-fold, 10 mM aqueous solution). In addition, similar experiments were conducted also in the presence of excess of metals such as ZnCl_2_, FeCl_3_ and CuCl_2_. In these cases, we evaluated if transmetallation occurs in presence of an excess (ca. 60-fold) of each metal (1 mM aqueous solution). We verified the behaviour of complexes over 13 days. In the case of FeCl_3_ and EDTA various level of decomplexation were observed after 216 h (Fig. 3).

**Fig. 4:**
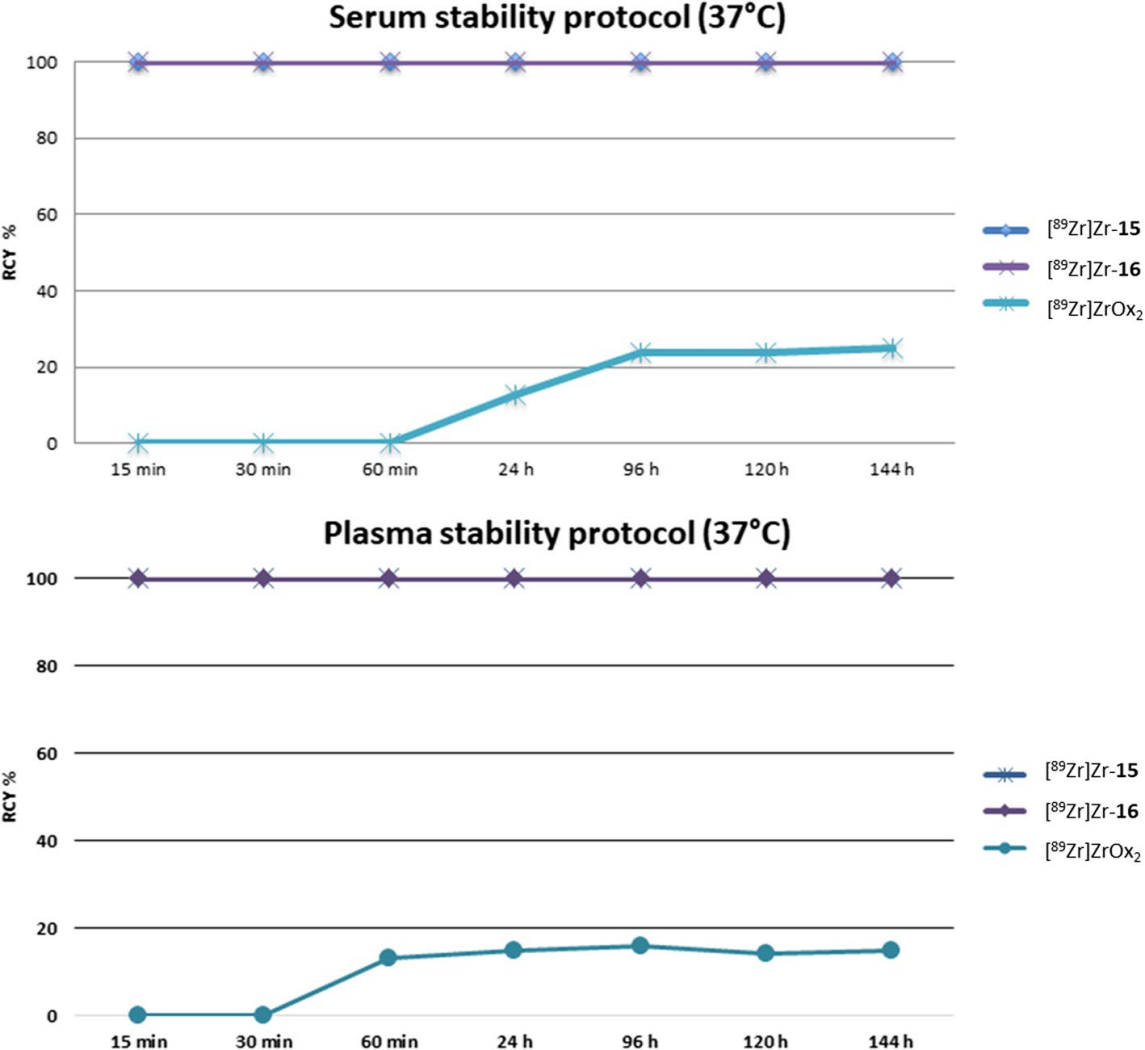
Results from stability studies on [^89^Zr]Zr-complexes in serum and plasma. The graph shows the values resulting from the iTLC used to study the stability of the complexes between **15** and **16** with [^89^Zr]ZrOx_2_ in serum and plasma

These encouraging results prompted us to verify the stability of the examined complexes in serum and plasma (Fig. 4): in both cases the samples were monitored by iTLC confirming a 100% complexation rate up to 6 days. The complexes were prepared by reaction of **15** and **16** (1.0 mg/mL in water) with [^89^Zr]Zr-oxalate (25.5 MBq, 100 µL) in 150 µL of 0.5 M HEPES. Completion of radiolabeling was established by iTLC and the resulting complexes were directly used for serum stability assays without further purification. The serum and plasma stability of the radiolabeled complexes were assessed by measuring the radiolabeling efficacy (%) vs complexation with serum and plasma proteins using iTLC 50 mM EDTA. [^89^Zr]ZrOx_2_ was incubated in the same conditions of complexes to evaluate the unspecific protein binding.

### Conformational models

The complexation ability and the solubility profile observed for **13** prompted us to investigate this ligand in more detail along with the tetrapeptide **14** (Scheme 2) that has the same chelating groups of **13** but the absolute configuration of two of the four aminoacidic residues is inverted. This structural modification was designed to variate the spatial disposition of binding groups, and consequently, to evaluate any consequent change in the chelating behaviour. In addition, we also studied the ligands **15** and **16** (Scheme 2), obtained through the introduction of structural elements (polyethylene glycol chains) that can improve both the water-solubility and the chain flexibility.

**Fig. 5:**
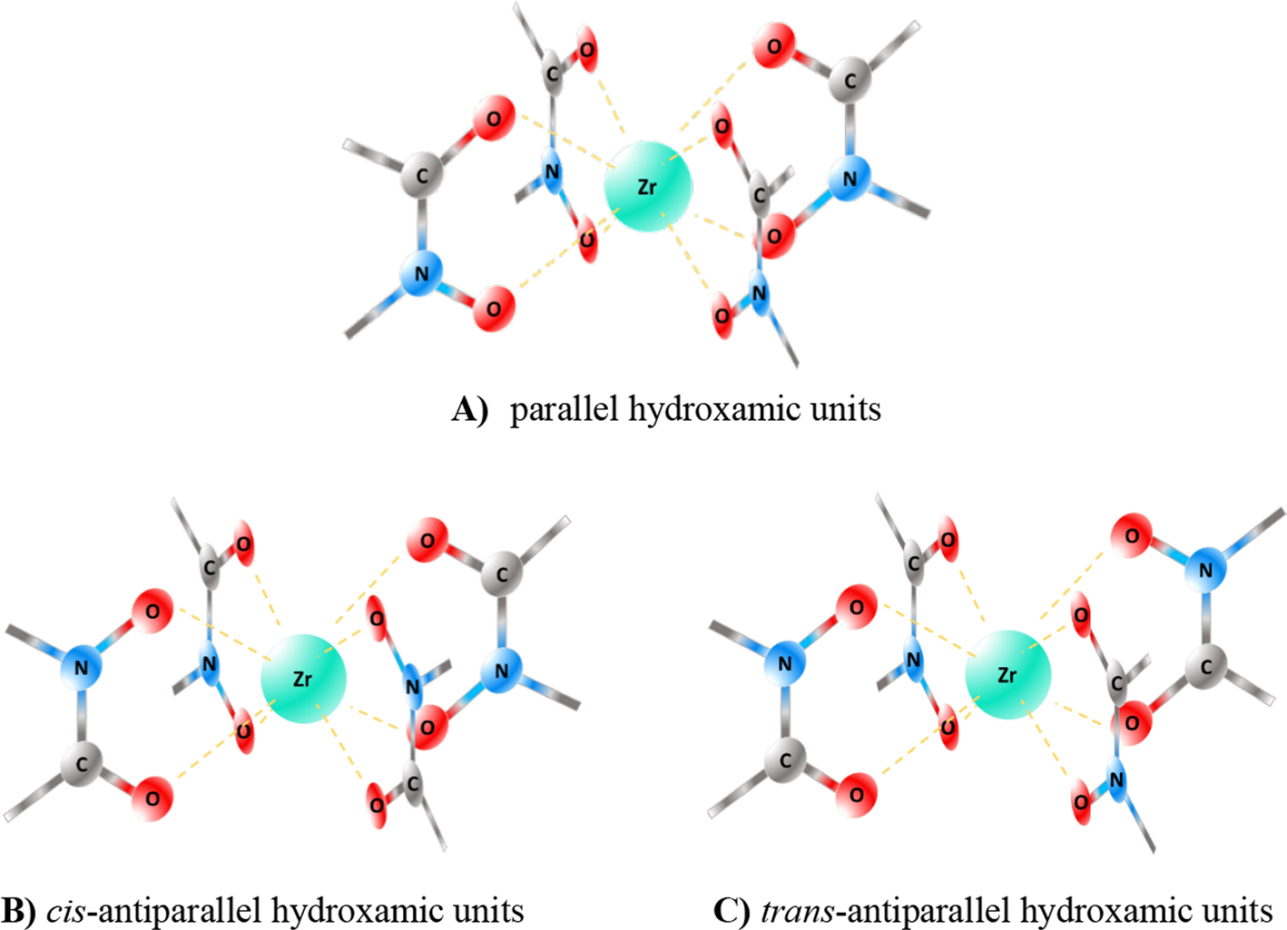
Parallel **A** and antiparallel **B**, **C** configurations considered in model construction. The figure illustrates configurations **A**–**C** surrounding the metal, as observed in the crystal structures outlined in the literature, thus presumed to be the most probable coordination geometries

The scope of these analyses was not to extract information on the relative energies of the models but rather to obtain general information on the flexibility and adaptability of the ligand to the coordination requrements of the metal center. Indeed, these investigations highlighted how the template of ligands may suffer from stringent geometric constraints with a resulting low chelating ability, especially for **13** and **14** which are of smaller dimensions. Also, we have focused our attention on the three different configurations around the metal reported in Fig. 5 A-C found in the crystal structures described in the literature and therefore considered the most likely coordination geometries (Guérard and Lee 2013).

**Fig. 6:**
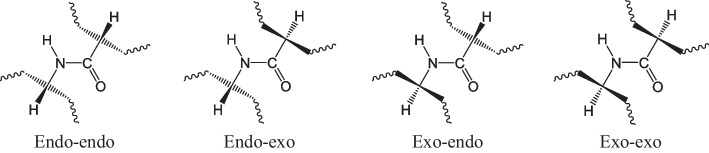
Conformations at the two chiral centres of **13** and **14.** The figure depicts four potential conformations at the two chiral centers linked to the central peptide bond of the scaffold. In these schematic representations, the Zr(IV) center is positioned above the plane of the peptide bond, facing toward the observer

Four possible conformations at the two chiral centres connected to the central peptide bond of the scaffold were considered, as represented in Fig. 6. In these schematic models the Zr(IV) centre is intended above the plane of the peptide bond, toward the observer. In the "exo" position the CH bonds point outwards, i.e. in the opposite direction to the metal centre, while the chains containing the coordinating groups point towards it. In the “endo” conformation the CH bonds point inside the complex which is formed, this means that they point towards the metallic centre, with the chains oriented towards the outside, which will wrap up to coordinate the metal.

**Fig. 7:**
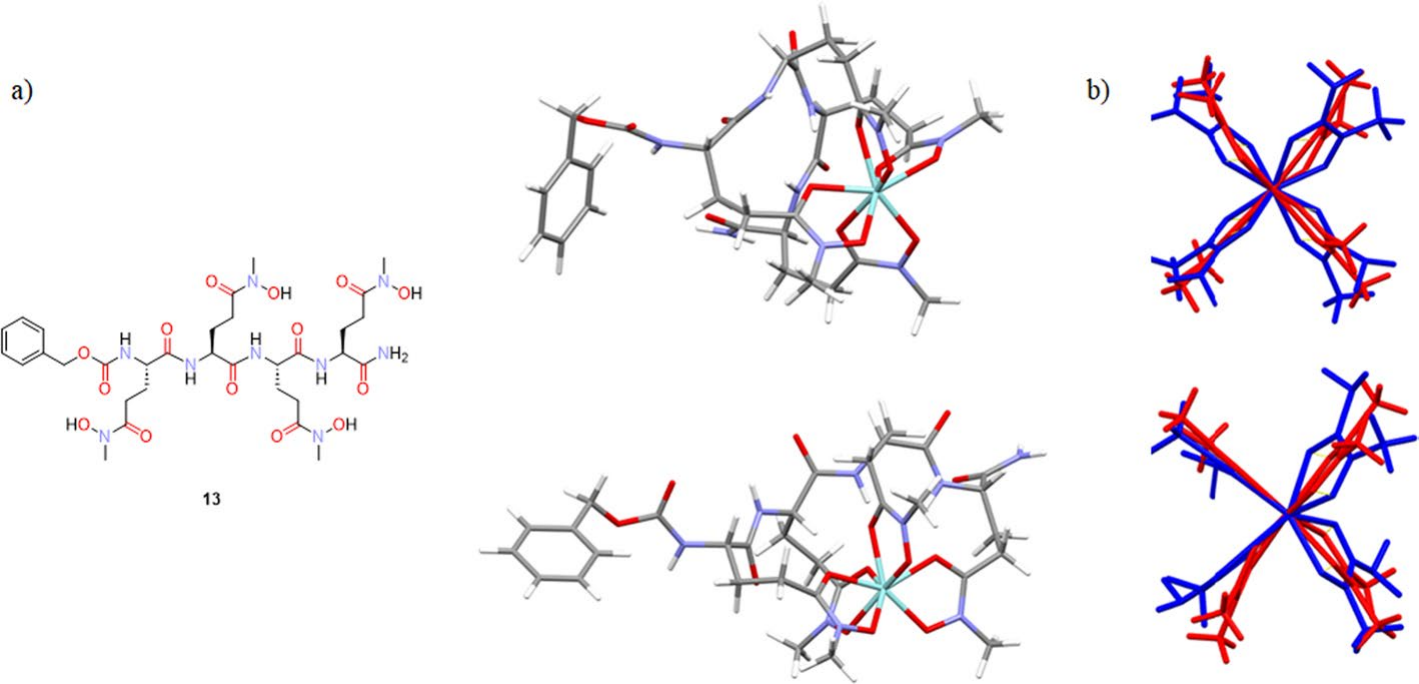
Three-dimensional optimized structures for ^nat^Zr-complex with **13**. The panel (**a**) shows three-dimensional optimized structures for ^nat^Zr-complex with **13** (depicted on the left as free ligand with the oxygen and nitrogen atoms coloured as in the neighbour 3D models) simulating exo-exo conformation (on the top) and the endo-exo disposition (on the bottom); section (**b**) depicts the overlay of the exo-exo conformation (blue) for ^nat^Zr-**13** with that reported in literature (red) on the top while on the bottom there is the overlapping for the endo-exo optimized structure for ^nat^Zr-**13** with the model from Brechbiel

The analysis of the models of the Zr(IV) complexes with **13** and **14** immediately highlighted that the length of the tetrapeptide scaffold allows the formation of only a complex with parallel arrangement of the hydroxamic units as in Fig. 5 A. Therefore, all the four different starting structures (exo/exo; exo/endo; endo/exo; endo/endo) were evaluated. We have qualitatively analysed the structures in terms of cis peptide bonds, and we investigated the presence of CH_2_-CH_2_ groups in eclipsed/staggered conformations to assess the relative stability of these configurations. None of the four different configurations (endo/exo) allowed for having at the same time all peptides in *trans* conformation, and all staggered C–C connections. Based on the structural similarity between the modelled Zr(IV) sites with that of the ^nat^Zr-acetohydroxamate complex reported by Guérard and Brechbiel (2013) we predict the exo-exo and endo-exo conformations for **13** as the most stable (Fig. 7). Based on the same criterion, the exo-exo and endo-endo conformations around the central peptide bond were predicted as the most stable for ^nat^Zr-**14** may be predicted.

A similar analysis was performed for the larger ligands **15** and **16**. Since the ligand’s backbone has a greater degree of flexibility we did not focus on the “exo” and “endo” conformations of the CH bonds in ^nat^Zr-**15** and ^nat^Zr-**16**. By virtue of this flexibility, all the (A-C) configurations of the hydroxamic groups at the Zr(IV) centre could be modelled for these molecules. Perhaps not surprisingly, for both ^nat^Zr-**15** and ^nat^Zr-**16** the best overlap between the chelation rings of the Zr(IV) centre and that of Zr-acetohydroxamate has been obtained for the antiparallel arrangement of the hydroxamates around the metal. However, while for ^nat^Zr-**16** the cis-antiparallel configuration is that presents the best overlap with the metal centre in Zr-acetohydroxamate (same *cis* configuration), the trans-antiparallel configuration presents the best overlap for ^nat^Zr-**15**. For all models, all ethylene groups in the arms were found in staggered conformation and no evident structural strains were observed.

**Fig. 8:**
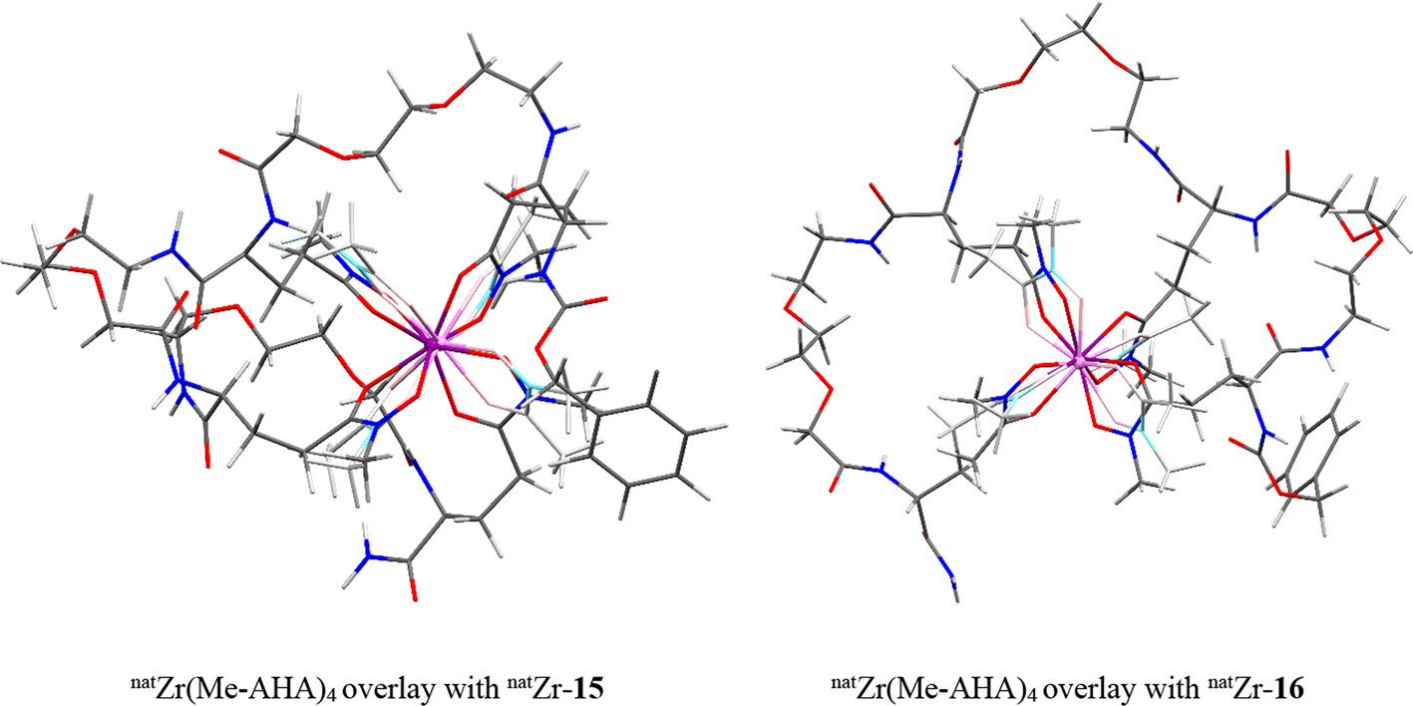
Model structures of **15** and **16** overlayed with ^nat^Zr(Me-AHA)_4_. The figure shows the overlap of two models of complexes ^nat^Zr-15 and ^nat^Zr-16 with that reported in literature for ^nat^Zr(Me-AHA)_4_. In **15** and **16**: zirconium in purple, oxygen in red, nitrogen in blue, carbon in gray. In ^nat^Zr(Me-AHA)_4_: zirconium in magenta, oxygen in pink, nitrogen in light blue, carbon in light gray

Figure 8 shows the overlap of two models of complexes ^nat^Zr-**15** and ^nat^Zr-**16** with that reported in literature for ^nat^Zr(Me-AHA)_4_ (Guérard and Lee 2013).

## Discussion

Combining solid phase and solution synthesis techniques, we prepared and studied novel ^89^zirconium-89-chelating molecules with a peptide scaffold. The adopted chemical design allowed modulation of molecular flexibility, hydrophilicity, and decoration with different zirconium chelating groups.

Experiments to evaluate the formation and stability of ^89^Zr-complexes were conducted using two different starting formulations: [^89^Zr]ZrCl_4_ and [^89^Zr]ZrOx_2_, which are common zirconium-89 formulations for radiopharmaceutical preparations. The [^89^Zr]ZrCl_4_ was mainly used to assess the chemical reactivity of the ligands at acidic pH, optimal for the complexation, but not ideal for the bioconjugation with monoclonal antibodies. In the same way, the relatively high temperature at which the radiolabeling reactions were performed (90 °C) was not compatible with the final conjugation step. On the contrary, studies with the oxalate-based precursor aimed to test the ligands within the pH and temperature ranges that are normally used for radiolabelling of monoclonal antibodies (i.e. with maximum temperatures of 37 °C and pH of 6–9). The initial results demonstrated intriguing chelating reactivity for the catechol ligand **12**, with quantitative conversion at room temperature and a pH of 3.5. This confirms that the catechol moiety is an interesting functional group for inclusion in zirconium-89 chelating development. Nevertheless, as detailed in Section “Evaluation of Zirconium-89 complexation and stability studies”, the exploration of the complex with the catecholate derivative **12** could not be further extended due to the occurrence of precipitate formation in most organic solvents. We were unable to clarify whether the generation of the insoluble species was due to the formation of the complex or to the occurrence of polymerization reactions. On the other hand, ligand **13**, based on *N*-methyl-substituted hydroxamic chelating moiety, showed slower reactivity under the same reaction conditions. Complete chelation was achieved at pH 3.5 after 60 min at room temperature or after 30 min at 50 °C. Both ligands -**12** and **13**- exhibited pure reactivity at high pH. The complexes obtained are stable under reaction conditions for over 6 days. Unfortunately, in challenge reactions, when competing with an excess of metals, the complexes did not exhibit high stability (See Additional file 1: Fig. S1).

[^89^Zr]ZrOx_2_ radiopharmaceutical precursor demonstrated compelling results under mild reaction conditions, achieving quantitative reaction at pH 7 within 15 min. The best results in terms of Zirconium-89 chelating properties were achieved with *N*-methyl hydroxamate moiety introduced as the side chain of a molecular scaffold composed of four aspartic acids alternated with polyethylene glycol units, as seen in compounds **15** and **16**. The labeling of compounds **15** and **16** was performed under reaction conditions suitable for bioconjugation (e.g. room temperature, water as solvent, neutral pH, short time of reaction, high yield). The kinetics of complex formation for **15** and **16** validated the capability of these molecules to rapidly coordinate the metal. This reactivity pattern is similar to the reference reactivity standard DFO and is compatible with ImmunoPET radiopharmaceutical preparations. As outlined in Section “Challenge experiments and in vitro stability of [89Zr]Zr-15 and [89Zr]Zr-16”, the complexes [^89^Zr]Zr-**15** and [^89^Zr]Zr-**16** were subjected to stability testing over time, including challenging reactions to assess stability against excess amounts of various metals such as copper, zinc, and iron. These assays were designed to simulate transmetallation conditions which can occur in vivo where the complexes are used in high dilution if compared to endogenous competing metals. Stability was also evaluated in the presence of excess EDTA, serum, and plasma. The water-soluble complexes [^89^Zr]Zr-**15** and [^89^Zr]Zr-**16** showed high resistance to transmetallation and stability in serum and plasma over 6 days.

The conformational investigations, as detailed in Section “Conformational models”, have highlighted how the template of ligands may suffer from stringent geometric constraints with a resulting low chelating ability, especially for **13** and **14** which are of smaller dimensions. Specifically, the outcome of these analyses indicates that, if the antiparallel configurations of the Zr(IV) centre represent the most stable arrangement of the hydroxamate groups, these can only be achieved with **15** and **16**, not with the shorter ligands. Moreover, structural strains (e.g. eclipsed conformations) were found unavoidably present in the models of **13** and **14**; this suggests that, for ligands of these dimension and length, a delicate balancing between the stability of the configuration and geometry of the metal centre and the strains in the ligand framework may be at the origin of a reduced stability of the complex compared to that with ligands of larger dimensions.

Considering the positive results obtained with **15**, we proceeded to synthesize **17** (Fig. 2), an analogue of **15** strategically modified at the N-terminal position with the introduction of a maleimide moiety which can be used for the functionalization of antibodies via thiol-Michael reaction. Preliminary results on the complex-formation kinetics of **17** with Zirconium-89 are reported in Additional file 1: Table S3: remarkably, the radiolabeling efficiency was already complete after 15 min at 37 °C. The reactivity of the maleimide-modified ligand **17** aligns with that of ligand **15**, making it suitable for antibody conjugation in future in vivo studies.

## Conclusions

In this study, a series of bifunctional octadentate peptide and pseudopeptide ligands for Zirconium-89 were developed with the aim of further functionalizing them with antibodies to create new radiolabeled compounds suitable for immunoPET imaging. Hydroxamate, N-methylhydroxamate, and catecholate moieties were investigated for their ability to complex radioactive Zirconium-89. N-methylhydroxamate demonstrated to be the most effective ^89^Zr-chelating group. Moreover, enhancing flexibility and hydrophilicity by incorporating polyoxyethylene groups between the hydroxamate units resulted in chelators (**15** and **16**) capable of rapidly forming stable and water-soluble complexes with ^89^Zr in very mild reaction conditions, which are essential for bioconjugation. Of note, [^89^Zr]Zr-**15** and [^89^Zr]Zr-**16** showed high resistance to transmetallation and stability in serum and plasma over 6 days in challenge experiments with EDTA as a competitor ligand and metal ions such as Fe^3+^, Zn^2+^, and Cu^2+^. Coupled with the mild reaction conditions employed for complex preparation, these ligands emerged as strong candidates for translational studies, warranting investigation into their behaviour under in vivo conditions. Moreover, the maleimide analogue **17**, suitable for bioconjugation, could further extend the application potential of this kind of ligands for theranostic purposes.

## Experimental details

### Materials and methods: chemistry

Standard Fmoc-protected amino acids, the resin for peptide synthesis and all the chemicals were purchased from Fluorochem, Enamine, and Sigma-Aldrich. Unless otherwise indicated, the amino acids used were L-amino acids. Peptide and pseudopeptide derivatives were prepared by solid phase synthesis on a Biotage® Siro II fully automated synthesizer using a 5 mL reactor vessel at 0.11 mM scale using 4-(2′,4′-dimethoxyphenyl-Fmoc-aminomethyl)-phenoxyacetamido-norleucyl-MBHA resin (Rink amide MBHA resin, substitution: 0.55 mmol/g). Coupling reactions were conducted at room temperature for 30 min, except for D-amino acids and Fmoc-O2Oc-OH that required 1 h for each reaction. Natural and non-natural amino acids and the polyoxyethilene compound were solubilised in *N*,*N*-Dimethylformamide (DMF) using a 0.5 M concentration; N,N′-diisopropylcarbodiimide (DIC), 1-hydroxybenzotriazole (HOBt), acetic anhydride (Ac_2_O) and *N-*methylmorpholine (NMM) were dissolved in the same solvent at concentrations of 1.09 M, 0.78 M, 0.5 M and 0.25 M respectively. A solution of 20% piperidine in dimethylformamide (DMF) was used to deprotect the Fmoc-group. Peptides were cleaved from the resin using the standard cleavage cocktail (95% TFA, 2.5% H_2_O, 2.5% triethylsilane) at room temperature for 3 h. After this time, peptides were treated with ice cold diethyl ether and the solid precipitated was isolated using centrifugation. Analytical RP-HPLC analyses of the final compounds were performed on a Beckman 126 reverse-phase HPLC (RP-HPLC) system equipped with a Beckman 168 UV–Vis detector (monitoring at 220 and 254 nm) using a XBridge® C18 column (4.6 × 150 mm, 5 μm particle size) at a flow rate of 0.7 mL/min with a linear gradient of solvent A (H_2_O + 0.1% TFA) and solvent B (CH_3_CN + 0.1% TFA) over 25 min. Analytical RP-HPLC determinations were reported as column retention time (t_R_) in minutes and all products showed a degree of purity > 95% at 220 and 254 nm. Peptides were purified on a reverse-phase Waters Prep 600 HPLC system equipped with a Jupiter column C18 (250 × 30 mm, 300 Å, 15 µm spherical particle size). Gradients used consisted of A (H_2_O + 0.1% TFA) and B (40% H_2_O in CH_3_CN + 0.1% TFA) at a flow rate of 20 ml/min. UV detection wavelength for semi-preparative HPLC was 220 nm. Reactions were monitored by thin layer chromatography on pre-coated plates of silica gel F_254_ aluminium foils (Merck, Darmstadt, Germany) at 254 nm wavelength. When indicated, intermediates were purified through silica gel flash chromatography (silica gel, 0.040–0.063 mm), using appropriate eluent mixtures. The mass spectra were recorded with an ESI MICROMASS ZMD 2000 dissolving the samples in a solution of H_2_O/CH_3_CN/TFA (40:60:0.1). Compounds **15**, **16** and their respective complexes with ^nat^Zr were also analysed by a high-resolution mass spectrometer (HRMS) Q Exactive™ Hybrid Quadrupole-Orbitrap™ (Thermo-Fisher Scientific) equipped with HESI-II (ESI). For the HRMS spectra the samples were analysed dissolving them in a mixture H_2_O/CH_3_OH 3:7 with 0.1% of formic acid. ^1^H NMR analyses were obtained at ambient temperature using a Varian 400 MHz spectrometer and were referenced to residual [1]H signals of the deuterated solvents respectively (δ H 7.26 for CDCl_3_; δ H 2.50 for DMSO; δ H 4.79 for D_2_O); the following abbreviations were used to describe the shape of the peaks: s: singlet; d: doublet; dd: double doublet; t: triplet; m: multiplet.

#### General solid phase-assisted synthesis for compounds 3a-d and 6a-c (Scheme 1)

Tetrapeptides **3a-d** and pseudopeptides **6a-c** were synthesized using a solid-phase approach with the conditions described in the Sect. 4.1 “Materials & methods”.

#### Liquid phase synthesis

The syntheses of compounds **10** and **22** are detailed in the Supporting Information.

##### Synthetic procedure for 12 (Scheme 2)

To a stirred solution of compound **10** (1.76 mmol, 16.0 eq) in DMF (5 mL) HATU (1.94 mmol, 17.6 eq) and DIPEA (1.94 mmol, 17.6 eq) were added at 0 °C. After 5 min, a solution of **3b** (0.11 mmol, 1.0 eq) in DMF and DIPEA (0.11 mmol, 1.0 eq) was added in a dropwise manner. Reaction mixture was warmed to room temperature and within one hour the ESI–MS spectrum showed the completion of reaction. The solvent was removed by evaporation. Then the crude material was solved in ethyl acetate and washed with 10% citric acid, 5% NaHCO_3_ aqueous solution and brine. **12** was obtained after deprotection in 100% TFA at 40 °C for four hours. The product was purified using semi-preparative RP-HPLC (white amorphous solid, 16% overall yield).

MS (ESI): m/z calcd for C_52_H_68_N_9_O_16_ [M + H]^+^ 1075.16, found 1074.95. t_R_ = 14.93.

1H NMR (400 MHz, DMSO-d_6_): δ 7.43–7.36 (m, 4H), 6.88–6.82 (m, 8H), 4.55–4.24 (m, 2H), 4.08–3.43 (m, 2H), 3.26–3.18 (m, 8H), 1.82–1.53 (m, 16H), 1.39–1.27 (m, 8H). ^13^C NMR (DMSO-d_6_): 176.17, 172.38, 172.11, 171.35, 171.30, 171.24, 148.77, 146.03, 145.48, 121.07, 120.99, 120.80, 120.12, 117.10, 54.72, 54.53, 53.44, 52.51, 41.86, 33.81, 32.67, 32.54, 32.41, 29.81, 24.22.

##### Synthetic procedure for 9 (Scheme 2)

To a solution of the tetracysteine **3a** (0.033 mmol, 1.0 eq) in H_2_O/CH_3_CN (500µL/1 mL) and **7** (0.14 mmol, 4.4 eq), 10 µL of a 5% aqueous solution of NaHCO_3_ were added. The reaction was monitored via ESI–MS, which showed the completion of the reaction after five minutes at room temperature. The volatiles were evaporated, and the crude was lyophilized. After lyophilization, compound **8** was deprotected using TFA. When the mass spectrum showed the completion of reaction, TFA was removed, and the residue was purified by preparative HPLC to give **9** (6% yield).

MS (ESI): m/z calcd for C_44_H_64_N_13_O_20_S_4_ [M + H]^+^ 1223.31, found 1222.72. t_R_ = 10.41.

##### Synthetic pathway for 13–17 (Scheme 2)

Peptide derivatives **13–14** and pseudopeptides **15–17** were obtained using the standard coupling reaction conditions with HATU e DIPEA. The procedure for obtaining **13** is detailed below and is representative of all the compounds of this section.

To a solution of Cbz-tetraGlu-NH_2_ (**6a**, 0.025 mmol, 1.0 eq) in DMF (2 mL), were added HATU (0.12 mmol, 4.8 eq) and DIPEA (0.12 mmol, 4.8 eq) at 0 °C. After five min, *N-*methylhydroxylamine hydrochloride dissolved in DMF (0.5 mL) and DIPEA (4.8 eq) was dropped into the solution. The reaction was warmed to room temperature and the ESI–MS analysis showed the presence of the product after 15 min of reaction. The DMF was removed under pressure, the residue was dissolved in H_2_O/CH_3_CN and the solution was purified via preparative HPLC.

13

31% yield (white solid). MS (ESI): m/z calcd for C_32_H_50_N_9_O_14_ [M + H]^+^ 784.80, found 784.90. t_R_ = 15.08.

^1^H NMR (400 MHz, DMSO-d_6_): δ 7.33–7.12 (m, 5H), 5.36 (s, 2H), 4.99–4.93 (m, 1H), 4.37–4.03 (m, 3H), 2.74–2.52 (m, 12H), 2.46–2.31 (m, 8H), 2.26–2.04 (m, 8H). ^13^C NMR (DMSO-d_6_): δ 176.17, 172.28, 172.11, 159.04, 136.99, 128.33, 128.26, 128.21, 128.18, 67.05, 53.57, 52.83, 51.86, 39.72, 30.21, 26.91, 26.56.

14

23% yield (white solid). MS (ESI): m/z calcd for C_32_H_50_N_9_O_14_ [M + H]^+^ 784.80, found 784.57. t_R_ = 12.77.

^1^H NMR (400 MHz, DMSO-d_6_): 7.37–7.20 (m, 5H), 5.35–5.17 (m, 2H), 4.52–4.39 (m, 4H), 2.68–2.50 (m, 12H), 2.26–2.04 (m, 16H). ^13^C NMR (DMSO-d_6_): 176.44, 172.30, 171.99, 159.15, 137.05, 128.29, 128.20, 128.17, 67.01, 53.72, 53.04, 51.78, 39.66, 30.47, 26.90, 26.61.

15

68% yield (pale yellow oil). HRMS (ESI +): m/z calcd for C_50_H_83_N_12_O_23_ [M + H]^+^ 1219.5689, found 1219.5682. t_R_ = 13.02.

^1^H NMR (600 MHz, D_2_O): δ 7.34–7.27 (m, 5H), 5.04–4.99 (m, 2H), 4.32–4.28 (m, 3H), 4.02–3.97 (m, 7H), 3.63–3.52 (m, 18H), 3.38–3.24 (m, 9H), 3.12–3.10 (m, 9H), 2.53–2.35 (m, 8H), 1.80–2.06 (m, 8H). ^13^C NMR (D_2_O): δ 175.83, 174.29, 174.25, 174.20, 174.12, 174.10, 173.15, 173.08, 172.74, 172.56, 172.52, 157.67, 136.32, 128.85, 128.48, 127.70, 70.56, 69.44, 68.87, 67.18, 52.98, 52.94, 52.56, 39.05, 36.03, 27.98, 26.62, 26.41.

16

61% yield (pale yellow oil). HRMS (ESI +): m/z calcd for C_50_H_83_N_12_O_23_ [M + H]^+^ 1219.5689, found 1219.5687. t_R_ = 10.71.

^1^H NMR (600 MHz, D_2_O): δ 7.33–7.27 (m, 5H), 5.04–5.00 (m, 2H), 4.30–4.25 (m, 3H), 4.01–3.97 (m, 7H), 3.61–3.51 (m, 18H), 3.37–3.25 (m, 9H), 3.10 (m, 9H), 2.54–2.34 (m, 8H), 1.77–2.06 (m, 8H). ^13^C NMR (D_2_O): δ 175.78, 174.11, 173.09, 173.01, 172.67, 172.50, 172.46, 157.67, 136.32, 128.78, 128.42, 127.66, 70.47, 69.36, 68.80, 67.14, 54.95, 52.94, 52.88, 52.52, 38.97, 37.05, 35.95, 29.98, 28.02, 27.91, 26.54, 26.34.

##### Synthesis of maleimide derivative 17

To a solution in DMF (2.0 mL) of **6d** (0.025 mmol, 1.0 eq) were added HATU (0.11 mmol, 4.4 eq) and DIPEA (4.4 eq) at 0 °C. After five minutes, *N*-methyl hydroxylamine was added, and the reaction mixture was warmed to room temperature. When reaction was completed, the solvent was evaporated, and the residue was purified via semi-preparative HPLC (pale yellow oil, 29% yield).

MS (ESI): m/z calcd for C_50_H_84_N_13_O_24_ [M + H]^+^ 1251.29, found 1251.46. t_R_ = 12.81.

##### Synthetic procedure for 18

HATU (0.16 mmol, 1.2 eq) and DIPEA (0.16 mmol, 1.2 eq) were added to a solution of the Lysine derivative **22** (“Synthetic procedures” section for the structure, 0.13 mmol, 1.0 eq) in DMF (2 mL) at 0 °C. After ten minutes, a previously prepared solution of DFO in DMF was added to the first one and the reaction was warmed to room temperature. After 15 min, the ESI–MS analysis showed the disappearance of the peak corresponding to the starting **22**. The solvent was evaporated, and the residue was treated with 4N HCl in dioxane solution (4 mL). The Boc-deprotection was followed by ESI–MS and, after completion, the crude was purified using semi-preparative HPLC (colourless oil, 17% overall yield).

MS (ESI): m/z calcd for C_39_H_69_N_9_O_12_ [M + 2H]^2+^ 428.02, found 428.13. t_R_ = 12.35.

##### Preparation of ^nat^Zr-complexes

Here it is described the general procedure for **15**/**16**-complexation. To a solution of the ligand **15** or **16** (8.2 µmol, 1.0 eq) in MeOH (3 mL) zirconium(IV) acetylacetonate (8.2 µmol, 1.0 eq) was added at room temperature. After 15 min, the solution was analysed via ESI–MS, which confirmed the formation of a 1:1 ligand/metal complex. The solvent was removed under pressure, and the residue was dissolved in a mixture of H_2_O/CH_3_CN and purified via preparative HPLC. The complex was then analysed via analytical HPLC and HRMS.

^nat^Zr-15

67% yield (colourless oil). HRMS (ESI +): m/z calcd for C_50_H_78_N_12_O_23_Zr [M + H]^+^ 1305.4423, found 1305.4430. t_R_ = 12.52.

^nat^Zr-16

55% yield (colourless oil). HRMS (ESI +): m/z calcd for C_50_H_78_N_12_O_23_Zr [M + H]^+^ 1305.4423, found 1305.4420. t_R_ = 12.07.

### Materials and methods: complexation protocols

^89^Zr was produced on an ACSI cyclotron (Advance Cyclotron System Inc, Richmond, Canada) using the automatized system Eckert e Ziegler (Francoforte, Germania) [40].

The radiolabeling of ligands was monitored using instant thin-layer chromatography paper (iTLC-SG) (Agilent Technologies, Santa Clara, California) using a 50 mM solution of EDTA as eluent and they were analysed on a Cyclone Plus radio-iTLC plate reader (PerkinElmer, Waltham, Massachusetts).

The HPLC used for the analyses of complexes consisted of a Shimadzu Prominence Modular HPLC equipped with an UV detector and a radioactive scanner Gabi (Raytest, Straubenhardt, Germany). The column used was a Luna Omega (5 μm Polar C18, 250 × 4,6 mm) and the analyses were conducted using the same solvents A and B described above for analytical HPLC.

All solutions used were prepared using metal free water purchased by Merck Company (Darmstadt, Germany).

#### Experimental for complexation and stability studies (chelation protocol)

[^89^Zr]Zr-DFO-Bz-NCS was prepared by the complexation of [^89^Zr]ZrCl_4_ (in 0.5 M HCl aq.) with DFO-Bz-NCS. Aliquots of [^89^Zr]Zr-chloride (250 µL, 75–300 MBq) were diluted in 150 µL of water (or 0.5 M HEPES). When the experiments were carried out at pH 7.0, the pH was adjusted neutralising the excess of aqueous HCl by the addition of the appropriate volume of K_2_CO_3_ (0.5 M in water). Chelator solution were prepared 1.0 mg/mL with DMSO (DFO-Bz-NCS). In a Eppendorf tube an aliquot of chelator 50 µL, (1.0 mg/mL in DMSO) was added to the appropriate solution of [^89^Zr]ZrCl_4_ and the reaction mixture was left to react at room temperature and 50 °C.

[^89^Zr]Zr-**9** was prepared by the complexation of [^89^Zr]ZrCl_4_ (in 0.5 M HCl aq.) with **9**. Aliquots of [^89^Zr]Zr-chloride (250 µL, 75–300 MBq) were diluted in 150 µL of water (or 0.5 M HEPES). When the experiments were carried out at pH 7.0, the pH was adjusted neutralising the excess of aqueous HCl by the addition of the appropriate volume of K_2_CO_3_ (0.5 M in water). Chelator solution were prepared 1.0 mg/mL with metal free water (**9**). In a Eppendorf tube an aliquot of chelator 50 µL, (1.0 mg/mL in water) was added to the appropriate solution of [^89^Zr]ZrCl_4_ and the reaction mixture was left to react at room temperature,50 °C and 90 °C.

[^89^Zr]Zr-**12** was prepared by the complexation of [^89^Zr]ZrCl_4_ (in 0.5 M HCl aq.) with **12**. Aliquots of [^89^Zr]Zr-chloride (250 µL, 75–300 MBq) were diluted in 150 µL of water (or 0.5 M HEPES). When the experiments were carried out at pH 7.0, the pH was adjusted neutralising the excess of aqueous HCl by the addition of the appropriate volume of K_2_CO_3_ (0.5 M in water). Chelator solution were prepared 1.0 mg/mL with water/DMSO 50/50 (**12**). In a Eppendorf tube an aliquot of chelator 50 µL, (1.0 mg/mL in water/DMSO 50/50) was added to the appropriate solution of [^89^Zr]ZrCl_4_ and the reaction mixture was left to react at room temperature, 50 °C and 90 °C.

[^89^Zr]Zr-**13** was prepared by the complexation of [^89^Zr]ZrCl_4_ (in 0.5 M HCl aq.) with **13**. Aliquots of [^89^Zr]Zr-chloride (250 µL, 75–300 MBq) were diluted in 150 µL of water (or 0.5 M HEPES). When the experiments were carried out at pH 7.0, the pH was adjusted neutralising the excess of aqueous HCl by the addition of the appropriate volume of K_2_CO_3_ (0.5 M in water). Chelator solution were prepared 1.0 mg/mL with metal free water (**13**). In a Eppendorf tube an aliquot of chelator 50 µL, (1.0 mg/mL in metal free water) was added to the appropriate solution of [^89^Zr]ZrCl_4_ and the reaction mixture was left to react at room temperature, 50 °C and 90 °C.

#### Experimental for complexation of 15, 16 and 17 with Zirconium-89, challenge and stability study

[^89^Zr]Zr-DFO-BzNCS was prepared by the complexation of [^89^Zr]Zr-oxalate (in 0.5 M oxalic acid) with DFO-Bz-NCS. Aliquots of [^89^Zr]Zr-oxalate (250 µL, 75–300 MBq) were diluted in 150 µL of water (or 0.5 M HEPES). The excess 0.5 M of oxalic acid was neutralized by adding the opportune volume of 0.5 M K_2_CO_3_ (aq). An aliquot of chelator (50 µL, 1.0 mg/mL in DMSO) was added to the solution of [^89^Zr]Zr-oxalate and the reaction mixture was left to react at room temperature and 50 °C.

[^89^Zr]Zr-**15**/**16** was prepared by the complexation of [^89^Zr]Zr-oxalate (in 0.5 M oxalic acid) with **15** or **16**. Aliquots of [^89^Zr]Zr-oxalate (250 µL, 75–300 MBq) were diluted in 150 µL of water (or 0.5 M HEPES). The excess 0.5 M of oxalic acid was neutralized by adding the opportune volume of 0.5 M K_2_CO_3_ (aq). An aliquot of chelator (50 µL, 1.0 mg/mL in metal free water) was added to the solution of [^89^Zr]Zr-oxalate and the reaction mixture was left to react at room temperature and 50 °C.

[^89^Zr]Zr-**17** was prepared by the complexation of [^89^Zr]Zr-oxalate (in 0.5 M oxalic acid) with **17**. Aliquots of [^89^Zr]Zr-oxalate (250 µL, 75–300 MBq) were diluted in 150 µL of water (or 0.5 M HEPES). The excess 0.5 M of oxalic acid was neutralized by adding the opportune volume of 0.5 M K_2_CO_3_ (aq). An aliquot of chelator (50 µL, 1.0 mg/mL in metal free water) was added to the solution of [^89^Zr]Zr-oxalate and the reaction mixture was left to react at room temperature and 37 °C.

#### Experimental for challenge of 15, 16 and 17 with Zirconium-89

At the solutions previous prepared 2.5 µL of different solution of CuCl_2_, FeCl_3_ and ZnCl_3_ (1 µmol/µL) was added to have 60-fold metal than ligand in study. Regarding the EDTA a solution of 1 µmol/µL was prepared on metal free water and 5 µL of the solution was added to the reaction mixture in order to have 120-fold EDTA.

#### Experimental for stability study of 15, 16 and 17 with Zirconium-89

[^89^Zr]Zr-**15**/**16**/**17** was prepared by the complexation of [^89^Zr]Zr-oxalate (in 0.5 M oxalic acid) with appropriate ligand. Aliquots of [^89^Zr]Zr-oxalate (250 µL, 75–300 MBq) were diluted in 150 µL of water (or 0.5 M HEPES). The excess 0.5 M of oxalic acid was neutralized by adding the opportune volume of 0.5 M K_2_CO_3_ (aq). An aliquot of chelator (50 µL, 1.0 mg/mL in metal free water) was added to the solution of [^89^Zr]Zr-oxalate and the reaction mixture was left to react at room temperature for 1 h, finally the reactivity was evaluated by iTLC. 25 µL of the reaction solution and of [^89^Zr]Zr-oxalate was add in a Eppendorf tube at 250 µL of Serum or Plasma and incubated at 37 °C.

### Materials and methods: conformational investigations

For each stating model the search of a conformation of the peptide corresponding to a local energy minimum was carried out by a simulated annealing procedure, using the software Hyperchem 8.0.7 (HyperCube Inc., 2009). The model was initially optimized using an MM + molecular mechanics force field. The model was then analyzed using molecular dynamics simulations by heating the molecule from 0 to 300 K (1 ps, 0.001 ps step size), then kept at 300 K for 1 ps, and then cooled down to 0 K. The molecular dynamics procedure was repeated two times.

### Supplementary Information

**Additional file 1.** Supplementary figures and tables.

## Data Availability

All data supporting the findings of this study are available within the paper and its Supplementary Information. The datasets used and/or analysed during the current study are also available from the corresponding author on reasonable request.

## Abbreviations

Ac_2_O: Acetic anhydride
AHA: Acetohydroxamate
BBS: Bombesin
BFCA: Bifunctional chelating agent
Boc: Tert-butyloxycarbonyl
Cbz: Benzyloxycarbonyl
Cys: Cysteine
DFO: Desferrioxamine
DIC: Diisopropylcarbodiimide
DIPEA: *N,N*-Diisopropylethylamine
DMF: *N,N*-Dimethylformamide
DOTA: 1,4,7,10-Tetraazacyclododecane-1,4,7,10-tetraacetic acid
EDTA: Ethylenediaminetetraacetic acid
Et_3_SiH: Triethylsilane
Fmoc: Fluorenylmethoxycarbonyl
Glu: Glutamic acid
HATU: Hexafluorophosphate azabenzotriazole tetramethyl uronium
HOBt: 1-Hydroxybenzotriazole
HOPO: Hydroxypyridinone
iTLC: Instant thin layer chromatography
Lys: Lysine
mAbs: Monoclonal antibodies
NMM: *N-*Methylmorpholine
O2Oc: 8-Amino-3,6-dioxaoctanoic acid
PET: Positron emission tomography
SPPS: Solid phase peptide synthesis
TFA: Trifluoroacetic acid
