# Supplementary material for: Bifunctional octadentate pseudopeptides as Zirconium-89 chelators for immuno-PET applications
Source: EJNMMI Radiopharm Chem. 2024 May 6;9:38. doi: 10.1186/s41181-024-00263-1 (PMC11070408; doi:10.1186/s41181-024-00263-1)
Supplement: Supplementary file 1 — Additional file 1. Supplementary figures and tables. [file 41181_2024_263_MOESM1_ESM.docx]

**Bifunctional octadentate pseudopeptides as Zirconium-89 chelators for immuno-PET applications**

Valentina Albanese^1^*, Chiara Roccatello^2^, Salvatore Pacifico^2^, Remo Guerrini^2^, Delia Preti^2^, Silvia Gentili^3^, Matteo Tegoni^3^, Maurizio Remelli^2^*, Denise Bellotti^2^, Jonathan Amico^4^, Giancarlo Gorgoni^4^, Emiliano Cazzola^4^

*^1^Department of Environmental and Prevention Sciences, University of Ferrara, Palazzo Turchi di Bagno, C.so Ercole I D'Este 32, 44121 Ferrara, Italy*

*^2^Department of Chemical, Pharmaceutical and Agricultural Sciences, University of Ferrara, Via Luigi Borsari 46, 44121 Ferrara, Italy*

*^3^Department of Chemistry, Life Sciences and Environmental Sustainability, University of Parma, Parco Area delle Scienze 11/A, 43124 Parma, Italy*

*^4^IRCCS Sacro Cuore Don Calabria Hospital, Via Don A. Sempreboni 5, 37024 Negrar di Valpolicella (Verona), Italy*

* Corresponding authors.

E-mail addresses: lbnvnt@unife.it (V. Albanese), rmm@unife.it (M. Remelli).

| **Table of Contents** | **Pag.** |
| --- | --- |
| Chemistry: Synthetic procedures | S1 |
| Table S1: Radiolabeling efficiency of [^89^Zr]Zr-DFO-Bz-NCS, [^89^Zr]Zr-Compd **9**, [^89^Zr]Zr-Compd **12** and [^89^Zr]Zr-Compd **13** starting from [^89^Zr]ZrOx_2_ | S3 |
| Table S2: Complexation result for [^89^Zr]Zr-**14** starting from [^89^Zr]ZrCl_4_, comparison with [^89^Zr]Zr-**13** | S3 |
| Figure S1: Stability and challenge result for [^89^Zr]Zr-**12**, [^89^Zr]Zr-**13** and [^89^Zr]Zr-**14** starting from [^89^Zr]ZrCl_4_. | S4 |
| Complexation studies: Complexation of **15** and **16** with ^nat^Zr | S6 |
| HPLC profiles of free ligands **13** and **15** | S10 |
| HPLC profiles of free ligand **15**, its complexes with ^nat^Zr and ^89^Zr and their overlaps | S11 |
| Table S3: Radiolabeling efficiency of [^89^Zr]Zr-Compd **17** | S12 |

**CHEMISTRY: Synthetic procedures**

*Synthetic procedure for 2,2-diphenyl-1,3-benzodioxole-4-carboxylic acid* *(****10****)*

*Synthesis of methyl 2,3-dihydroxybenzoate* *(****19****).* Compound **19** was obtained with a procedure previously reported in literature.^1^ To a suspension of 2,3-dihydroxybenzoic acid (3.24 mmol, 1.0 eq) in MeOH (6 mL), 125 µL of concentrated sulfuric acid were added. The reaction was heated at 65 °C and left to reflux overnight under stirring. The completion of the reaction was monitored via ESI-MS. The solvent was removed under vacuum and the residue was portioned between water (25 mL) and ethyl acetate (25 mL). The aqueous layer was extracted with ethyl acetate three times before drying with anhydrous Na_2_SO_4_. The product was purified via column chromatography using a 4:1 mixture of petroleum ether and ethyl acetate as eluent. The desired product was obtained as a white solid (84% yield) for which NMR data matched with those previously reported in literature.^2^

*Synthesis of methyl 2,2-diphenylbenzo[d][1,3]dioxole-4-carboxylate (****20****).* To methyl 2,3-dihydroxybenzoate (2.40 mmol, 1.0 eq) was added dichlorodiphenylmethane (3.60 mmol, 1.5 eq) and the mixture was warmed at 160 °C for 1 hour. The reaction was monitored via ESI-MS, which showed the presence of the desired compound in addition to a by-product, the benzophenone. After one hour, the crude was diluted with ethyl acetate (25 mL) and washed with a saturated aqueous solution of NaHCO_3_ (10 mL) and brine (10 mL). The organic layer was dried using anhydrous Na_2_SO_4_ and the solvent was evaporated under reduced pressure. After crystallization from MeOH a solid was isolated as an inseparable mixture of the desired product and the by-product that was bused in the next step without further purification.

*Synthetic procedure for 2,2-diphenyl-1,3-benzodioxole-4-carboxylic acid (****10****).* The mixture obtained from the previous step was dissolved in a 1:1 mixture of MeOH and NaOH 1M (20 mL). The reaction was refluxed and monitored via ESI-MS which showed the completion of reaction after 3 hours. The mixture was first neutralised using a 10% solution of acetic acid and then extracted with ethyl acetate for three times. The crude was chromatographed using petroleum ether/ethyl acetate 4:1 giving the pure product as white solid (75 % yield). The spectral properties were comparable with which previously reported in literature.^2^

*Synthesis of N-(tert-butoxy)-4-(2,5-dioxo-2,5-dihydro-1H-pyrrol-1-yl)butanamide (****7****).* 4-(2,5-dioxo-2,5-dihydro-1H-pyrrol-1-yl)butanoic acid was prepared as previously described in literature.^3-5^ This compound (0.36 mmol, 1.1 eq) was dissolved in DMF (2 mL) and the solution was cooled at 0 °C. Then HATU (0.36 mmol, 1.1 eq) and DIPEA (0.36 mmol, 1.1 eq) were added. After 5 minutes O-*tert*butyl hydroxylamine (0.33 mmol, 1.0 eq) was added to the solution and the reaction was warmed to room temperature. The ESI-MS spectrum confirmed the completion of reaction after 15 minutes. After evaporation of the solvent, the mixture was dissolved in ethyl acetate and washed with 10% citric acid, 5% NaHCO_3_ aqueous solution and brine. The organic phase was dried with anhydrous Na_2_SO_4_ and the ethyl acetate was removed by rotary evaporation. The desired product was purified through column chromatography (4:1 ethyl acetate/petroleum ether) that furnished **7** with 52 % yield (white solid).

MS (ESI): m/z calcd for C_12_H_19_N_2_O_4_ [M+H]^+^ 255.29, found 255.30. t_R_ = 12.98

^1^H NMR (400 MHz, CDCl_3_): δ 7.46 (s, 2H), 3.20 (t, J = 7.6 Hz, 2H), 2.22 (t, J = 5.7 Hz, 2H), 1.90-1.77 (m, 2H).

*Synthesis of N^2^-(tert-butoxycarbonyl)-N^6^-(4-(2,5-dioxo-2,5-dihydro-1H-pyrrol-1-yl)butanoyl)-L-lysine (****22****).* To a solution of Boc-Lys-OH (0.20 mmol, 1.0 eq) in DMF (2 mL) the O-activated succinimide ester of γ-aminobutyric maleimide **21** (0.20 mmol, 1.0 eq) and DIPEA (0.20 mmol, 1.0 eq) were added at room temperature. After two hours, the ESI-MS spectrum showed the presence of the product. The solvent was removed under vacuum, the crude was dissolved in ethyl acetate, extracted with 10% citric acid and brine. The organic layer was dried using anhydrous Na_2_SO_4_. The residue obtained after removal of the solvent was used in the next step without further purification.

MS (ESI): m/z calcd for C_19_H_30_N_3_O_7_ [M+H]^+^ 412,46, found 412.72. t_R_ = 13.60

**References**

[1] Captain, I., G. J. P. Deblonde, et al. (2016). "Engineered Recognition of Tetravalent Zirconium and Thorium by Chelator–Protein Systems: Toward Flexible Radiotherapy and Imaging Platforms." Inorganic Chemistry 55(22): 11930-11936.

[2] Weitl, F. L. and Raymond, K. N. (1980). “Specific sequestering agents for the actinides. 3. Polycatecholate ligands derived from 2,3-dihydroxy-5-sulfobenzoyl conjugates of diaza- and tetraazaalkanes” Journal of the American Chemical Society, 102(7): 2289–2293.

[3] Lambert, T. H., Danishefsky S. J. (2006). “Total Synthesis of UCS1025A.” J. Am. Chem. Soc., 128(2):426-427.

[4] Song, H. Y., Ngai, M. H., et al. (2009). “Practical synthesis of maleimides and coumarin-linked probes for protein and antibody labelling via reduction of native disulfides.” Organic & biomolecular chemistry, 7(17), 3400–3406.

[5] Sinclair, A. J., del Amo, V., & Philp, D. (2009). “Structure-reactivity relationships in a recognition mediated [3+2] dipolar cycloaddition reaction.” Organic & biomolecular chemistry, 7(16), 3308–3318.

***Supplementary Table S1***. Radiolabeling efficiency of [^89^Zr]Zr-DFO-Bz-NCS, [^89^Zr]Zr-Compd **9**, [^89^Zr]Zr-Compd **12** and [^89^Zr]Zr-Compd **13** starting from [^89^Zr]ZrOx_2_.

|  | |  | |  | |  | | |  |  |  |  |  |  |  |  |  |  |  |  |  |  |
| --- | --- | --- | --- | --- | --- | --- | --- | --- | --- | --- | --- | --- | --- | --- | --- | --- | --- | --- | --- | --- | --- | --- |
|  | **Time** | |  | |  | |  | | | |  | |  |  |  |  |  |  |  |  |  |  |
|  | | | **[^89^Zr]Zr-DFO-Bz-NCS** | | | | | | | **[^89^Zr]Zr-Compd 9** | | | | | **[^89^Zr]Zr-Compd 12** | | | **^89^Zr]Zr-Compd 13** | | | |  |
|  |  |  | **RT** | | **50 °C** | | | **RT** | | | | **50 °C** | | **90 °C** | **RT** | **50 °C** | **90 °C** | | **RT** | **50 °C** | **90 °C** | |
| **pH 3.5** | 15 min | | 100 | | 100 | | | 0 | | | | 0 | | NA | 80 | 85 | NA | | 45 | 52 | NA | |
|  | 30 min | | 100 | | 100 | | | 7 | | | | 5 | | NA | 85 | 97 | NA | | 55 | 72 | NA | |
|  | 60 min | | 100 | | 100 | | | 7 | | | | 10 | | NA | 95 | 100 | NA | | 100 | 100 | NA | |
| **pH 7.0** | 15 min | | 80 | | 79 | | | 25 | | | | 33 | | NA | 23 | 32 | NA | | 10 | 41 | NA | |
|  | 30 min | | 100 | | 100 | | | 35 | | | | 45 | | NA | 36 | 52 | NA | | 25 | 35 | NA | |
|  | 60 min | | 100 | | 100 | | | 40 | | | | 45 | | NA | 33 | 15 | NA | | 30 | 30 | NA | |

***Supplementary Table S2****.* Complexation result for [^89^Zr]Zr-**14** starting from [^89^Zr]ZrCl_4,_ comparison with [^89^Zr]Zr-**13**_._

|  | | **[^89^Zr]Zr-Compd 13** | | | **[^89^Zr]Zr-Compd 14** | | | |
| --- | --- | --- | --- | --- | --- | --- | --- | --- |
| **Time** | | **RT** | **50 °C** | **90 °C** | **RT** | **50 °C** | **90 °C** |  |
| **pH 3.5** | 15 min | 20 | 52 | 22 | 20 | 55 | 14 |  |
|  | 30 min | 33 | 100 | 16 | 20 | 73 | 22 |  |
|  | 60 min | 100 | 100 | 23 | 90 | 100 | 24 |  |
| **pH 7.0** | 15 min | 25 | 52 | 17 | 0 | 27 | 10 |  |
|  | 30 min | 31 | 26 | 10 | 24 | 42 | 15 |  |
|  | 60 min | 39 | 37 | 10 | 30 | 38 | 15 |  |

***Supplementary Figure S1***. Stability and challenge result for [^89^Zr]Zr-**12**, [^89^Zr]Zr-**13** and [^89^Zr]Zr-**14** starting from [^89^Zr]ZrCl_4_.

The graph shows the values resulting from the iTLC used to study the complexation and stability of the complexes [^89^Zr]Zr-**12,** [^89^Zr]Zr-**13** and [^89^Zr]Zr-**14**, measured in duplicate using iTLC eluted with a mobile phase consisting of 50 mM EDTA. From top to bottom: complexes formation and stability (Panel A); [^89^Zr]Zr-**12** stability in presence of competing metals (Panel B); [^89^Zr]Zr-**13** stability in presence of competing metals (Panel C); [^89^Zr]Zr-**14** stability in presence of competing metals (Panel D).

**COMPLEXATION STUDIES:** Complexation of **15** and **16** with ^nat^Zr

A 1:1 solution was prepared mixing zirconium acetylacetonate in methanol with **15**. After fifteen minutes at room temperature, the ESI-MS spectrum indicated the presence of a mono charged species with an *m/z* value matching with the protonated mono nuclear complex [Zr(**15**) + H]^+^ (calculated *m/z* = 1307.47) and [Zr(**15**) + 2H]^2+^ (calculated *m/z* = 654.24). No signal corresponding to the free ligand was detected.

The complex, prepared as described above, was then purified via semi-preparative HPLC to be characterized by analytical HPLC (Figure S2) and high-resolution mass spectrometry (HRMS, Figure S3).

Noteworthy, in the same HPLC experimental conditions, **15** and **16** showed a lower retention time respect to compound **13** confirming their marked hydrophilicity (see SI “HPLC chromatograms”, Supplementary Figures S7 and S8).


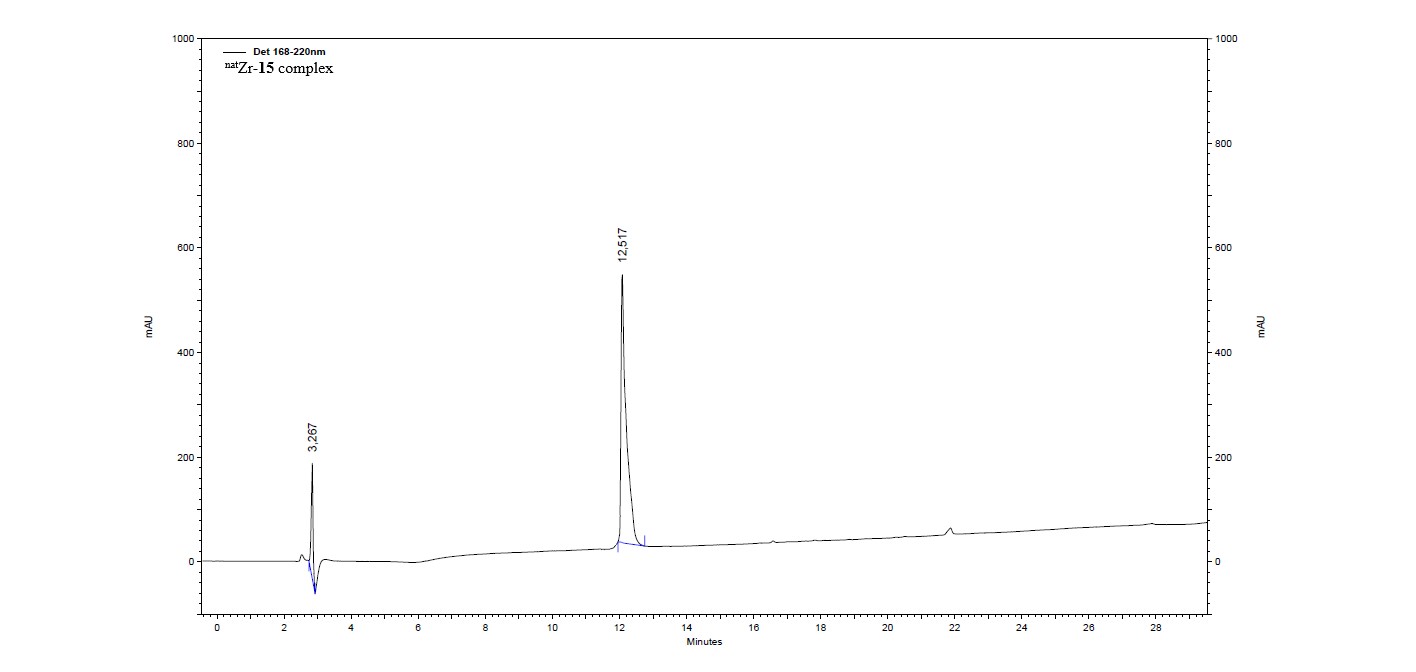


***Supplementary Figure S2***. Analytical profile of the ^nat^Zr-**15** complex after purification.

| 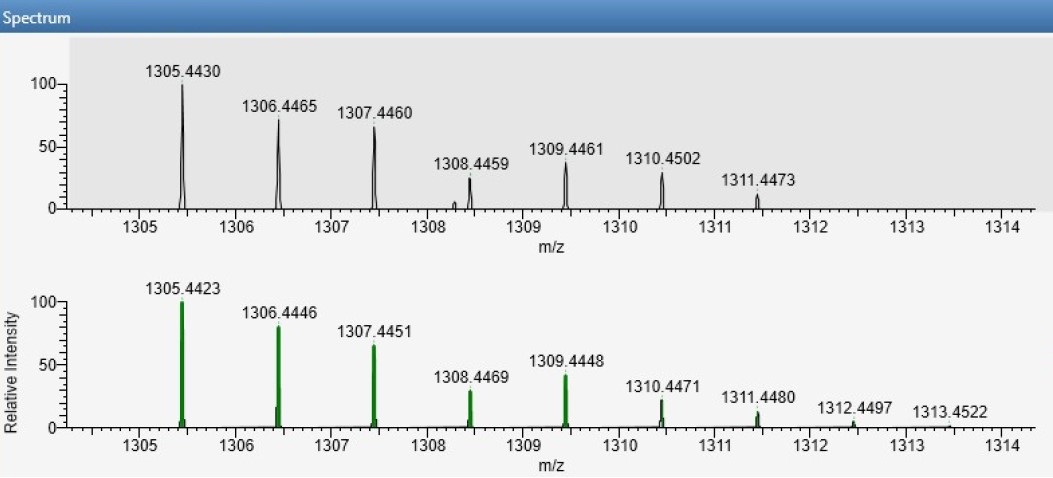 |
| --- |
| ***Supplementary Figure S3***. HRMS spectrum of the ^nat^Zr-**15** complex (top panel) and its isotopic-pattern simulation (bottom panel). |

The same complexation protocol with ^nat^Zr has been applied to **16** leading to comparable results as shown in Figures S4 and S5.


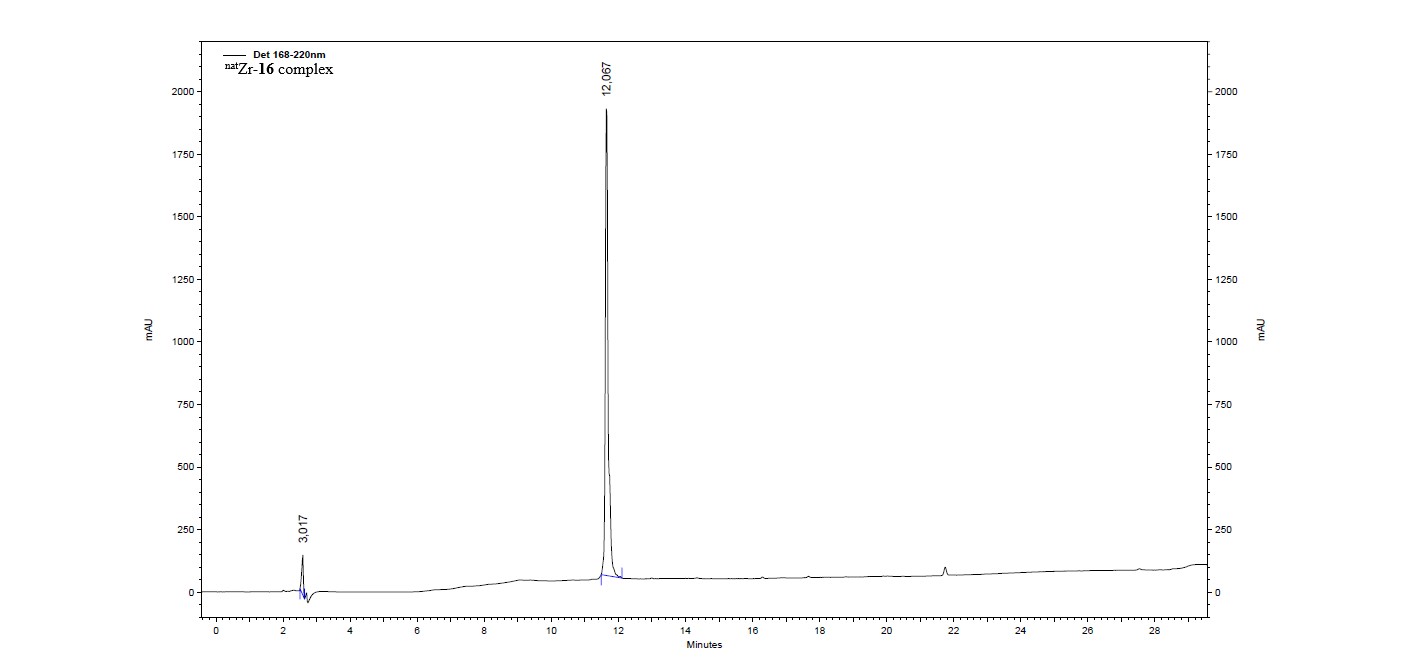


***Supplementary Figure S4****.* Analytical HPLC of the ^nat^Zr-**16** complex after semi-preparative HPLC purification.

| 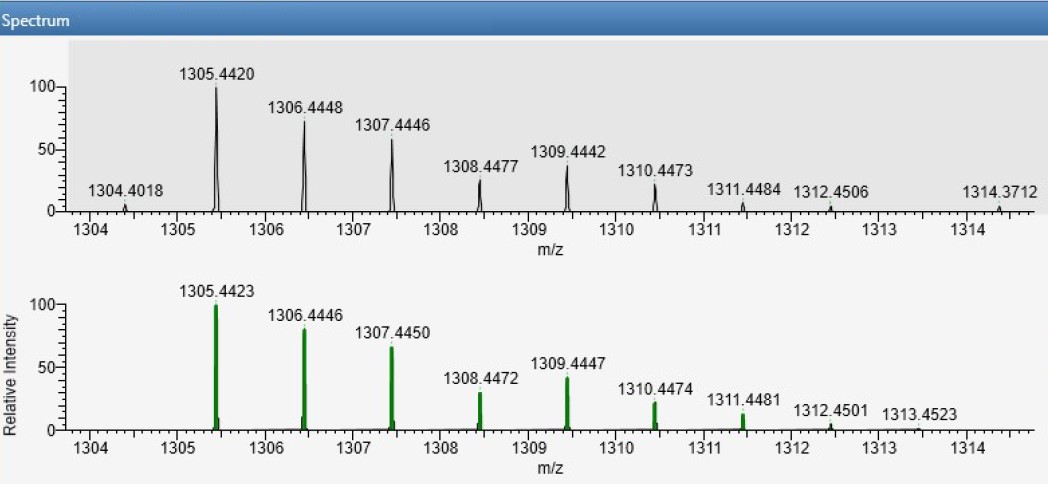 |
| --- |
| ***Supplementary Figure S5***. HRMS spectrum of the ^nat^Zr-**16** complex (top panel) and its isotopic-pattern simulation (bottom panel). |

With the aim to explore the complex formation kinetics using the cold metal, we also performed an ^1^H-NMR spectroscopic study on the ligands **15** and **16**. Since the results were very similar for the two compounds, the results will be described below only for **15**. The ligand was dissolved in deuterium oxide and the spectrum was first recorded at room temperature in the absence of zirconium (bottom trace in Figure S6). ZrCl_4_ was then added to the solution and the effect of the complexation reaction on ^1^H signals was monitored recording new spectra after 3 (middle trace in Figure S6) and 30 minutes (upper trace in Figure S6).

***Supplementary Figure S6*.** Overlayed amplified regions of the ^1^H-NMR spectra recorded for **15** as free ligand (below) or after 3 (in the middle) and 30 minutes (on the top) from the addition of ^nat^ZrCl_4_.

Comparing the traces reported in Figure S6, it is possible to observe a significant modification of the group of signals centred at 3.1 ppm for the ligand alone that was shifted to about 3.3 ppm in the spectrum recorded after 3 minutes from the addition of ZrCl_4_. These signals can be attributed to the protons of three *N*-methyl groups of hydroxamates. We assumed that coordination of Zr^4+^ resulted in a marked local effect on the shift of these proton groups spatially nearby to the hydroxyl substituents involved in coordinating zirconium. It has also to be considered that, upon reaction with Zr^4+^, the shapes of all the signals were broader than those observed with the free ligand, presumably because of the high flexibility of the molecule in complexing ^nat^Zr. Nevertheless, we cannot exclude that the low signals resolution may be attributed to the possibility of aggregate formation upon complexation. In addition, the pH value of the solution containing the ligand alone and that of the solution once the complexation was completed were measured, showing a change towards a more acidic pH in the latter case which was consistent with the formation of ^nat^Zr-**15** complex.

Moreover, the isolation of the ^nat^Zr-**15** complex made it possible to compare the HPLC profile of the latter with that of [^89^Zr]Zr-**15** radioactive complex (See Figure S9).

**HPLC CHROMATOGRAMS**

***Supplementary Figure S7.*** HPLC profile of compound **13**

**
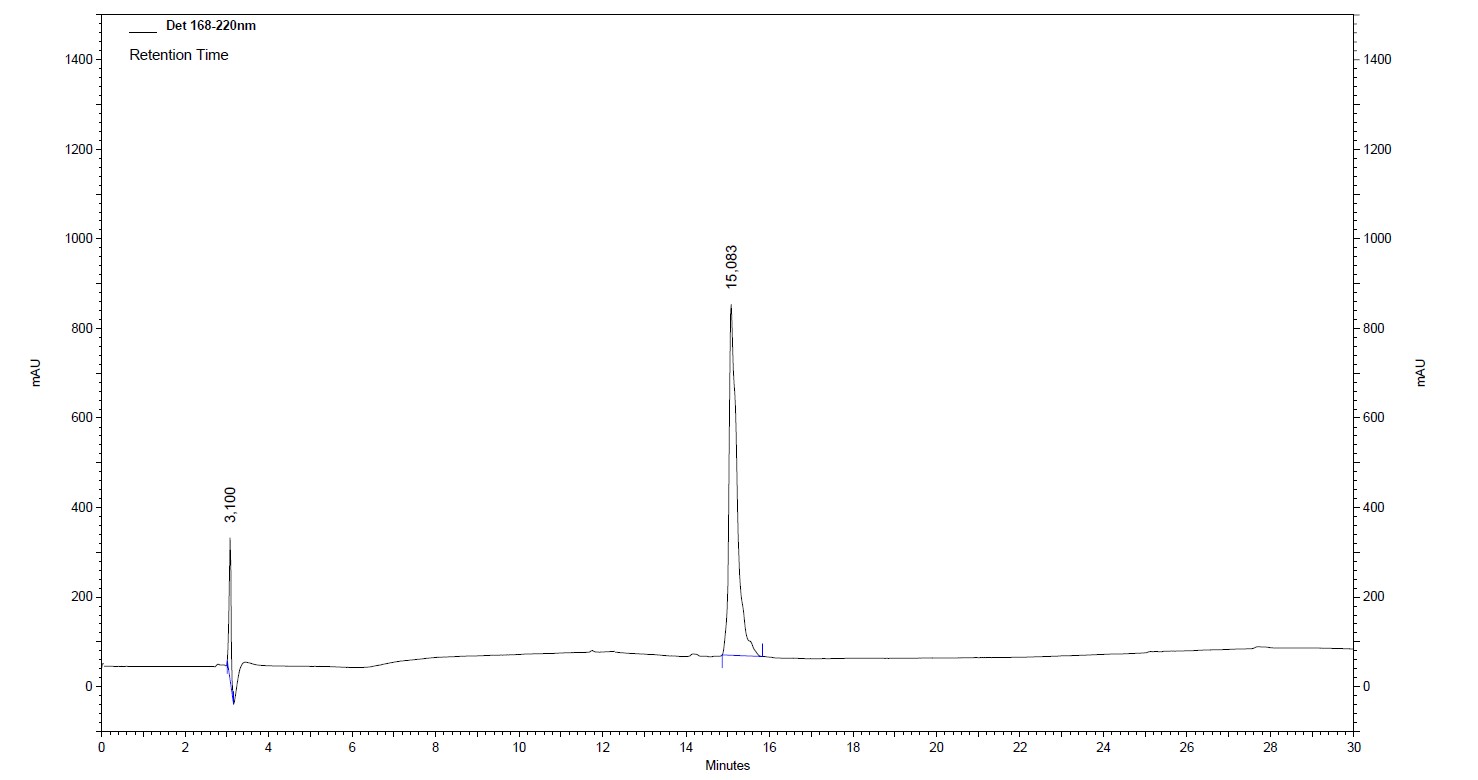
**

***Supplementary Figure S8.*** HPLC profile of compound **15**

***
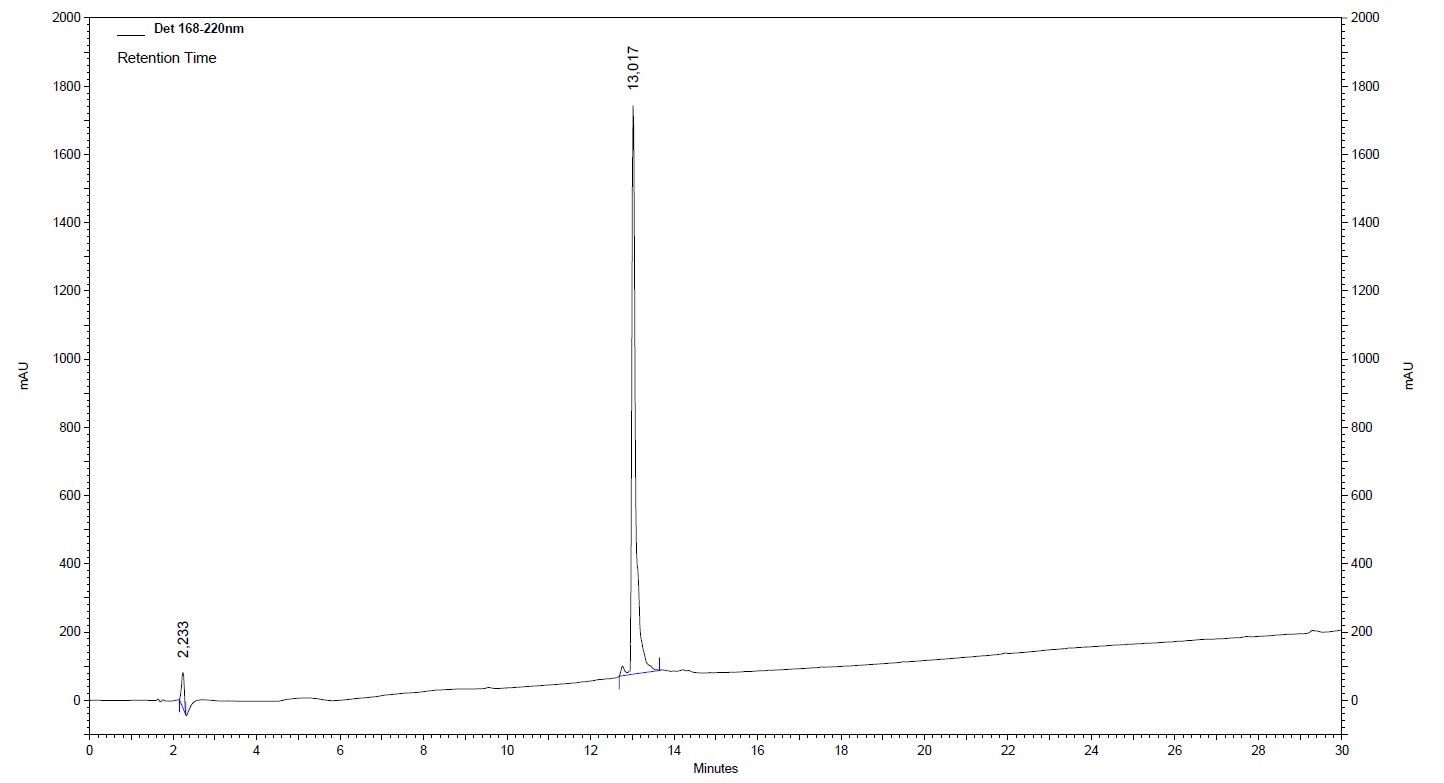
***

***Supplementary Figure S9***: HPLC profiles of free ligand **15**, its complexes with ^nat^Zr and ^89^Zr and their overlaps. From top to bottom the following chromatograms are reported: **a.** free ligand **15** (UV detection at 254 nm, t_R_= 19.7 min); **b.** ^nat^Zr-**15** complex (UV detection at 254 nm, t_R_= 20.4 min); **c.** co-injection ^nat^Zr-**15** and [^89^Zr]Zr-**15** (radioactive chromatogram t_R_= 20.2 min); **d.** co-injection ^nat^Zr-**15** and [^89^Zr]Zr-**15** (UV detection at 254 nm t_R_= 20.3); **e.** [^89^Zr]Zr-**15** radioactive chromatogram t_R_= 20.3 min). The chromatograms confirm that the retention times of the two complexes ^nat^Zr-**15** and [^89^Zr]Zr-**15** are superimposable. This procedure allow us to confirm the identity of the [^89^Zr]Zr-**15** that cannot be characterized by normal chemical procedure due to molar concentration of the complex present in solution.


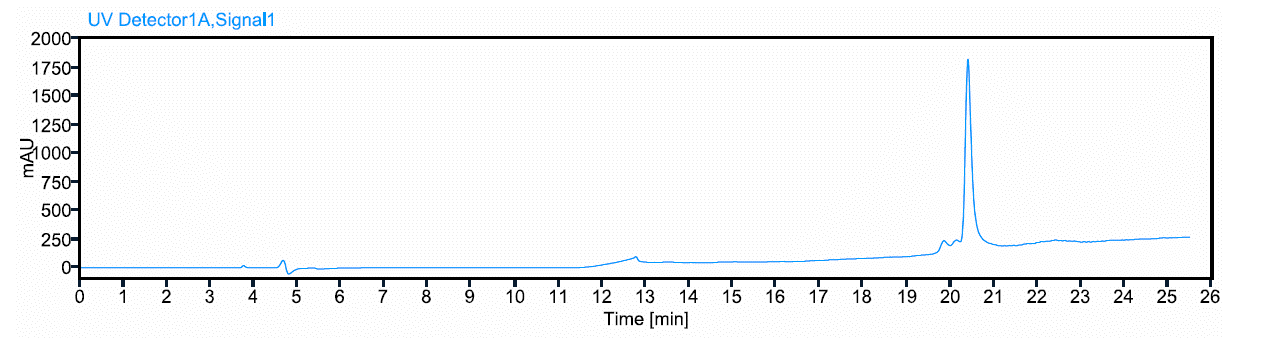

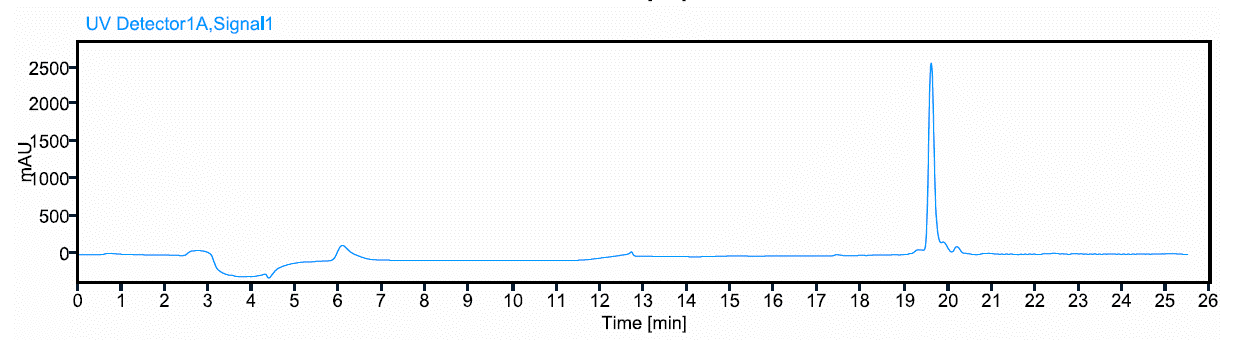

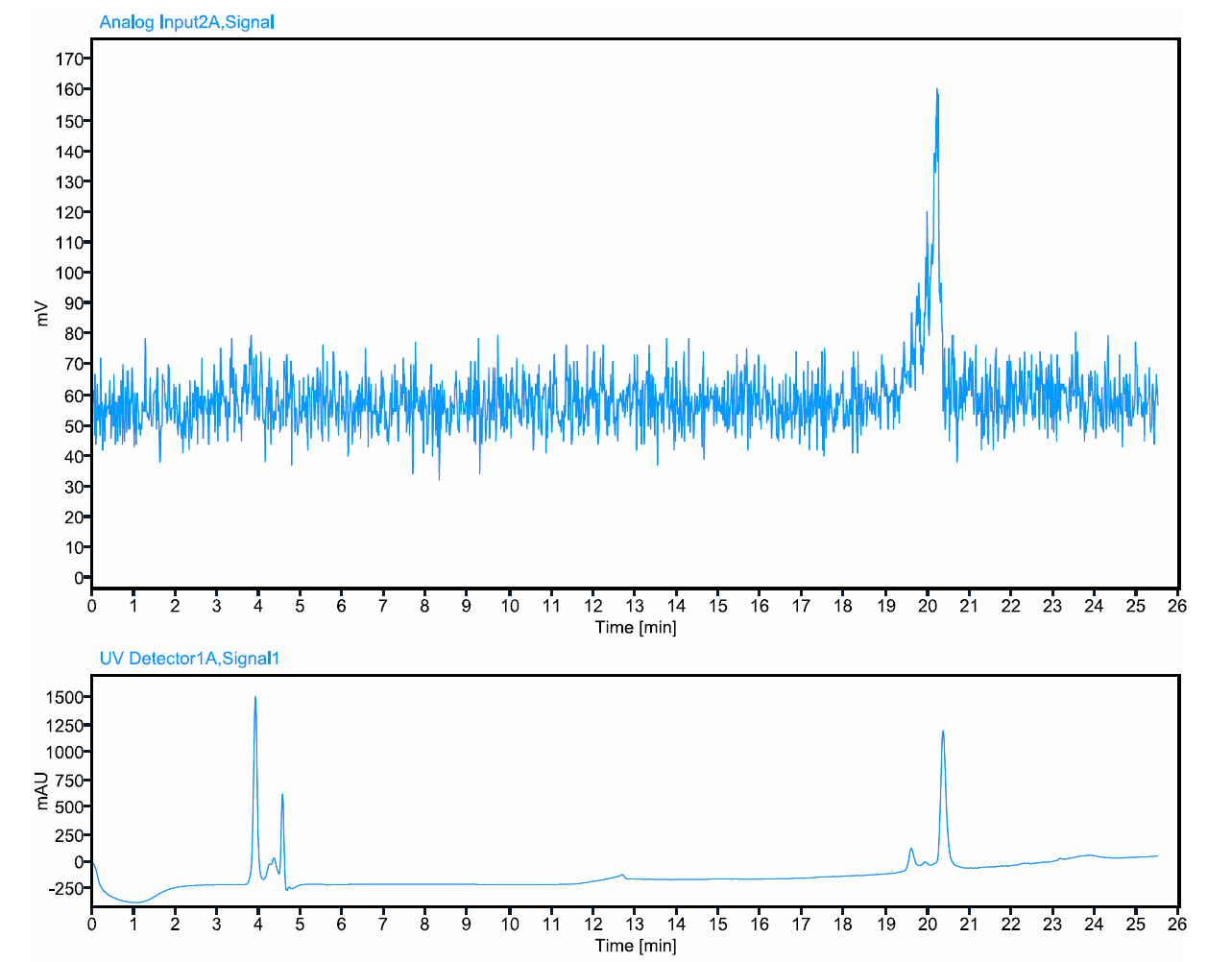

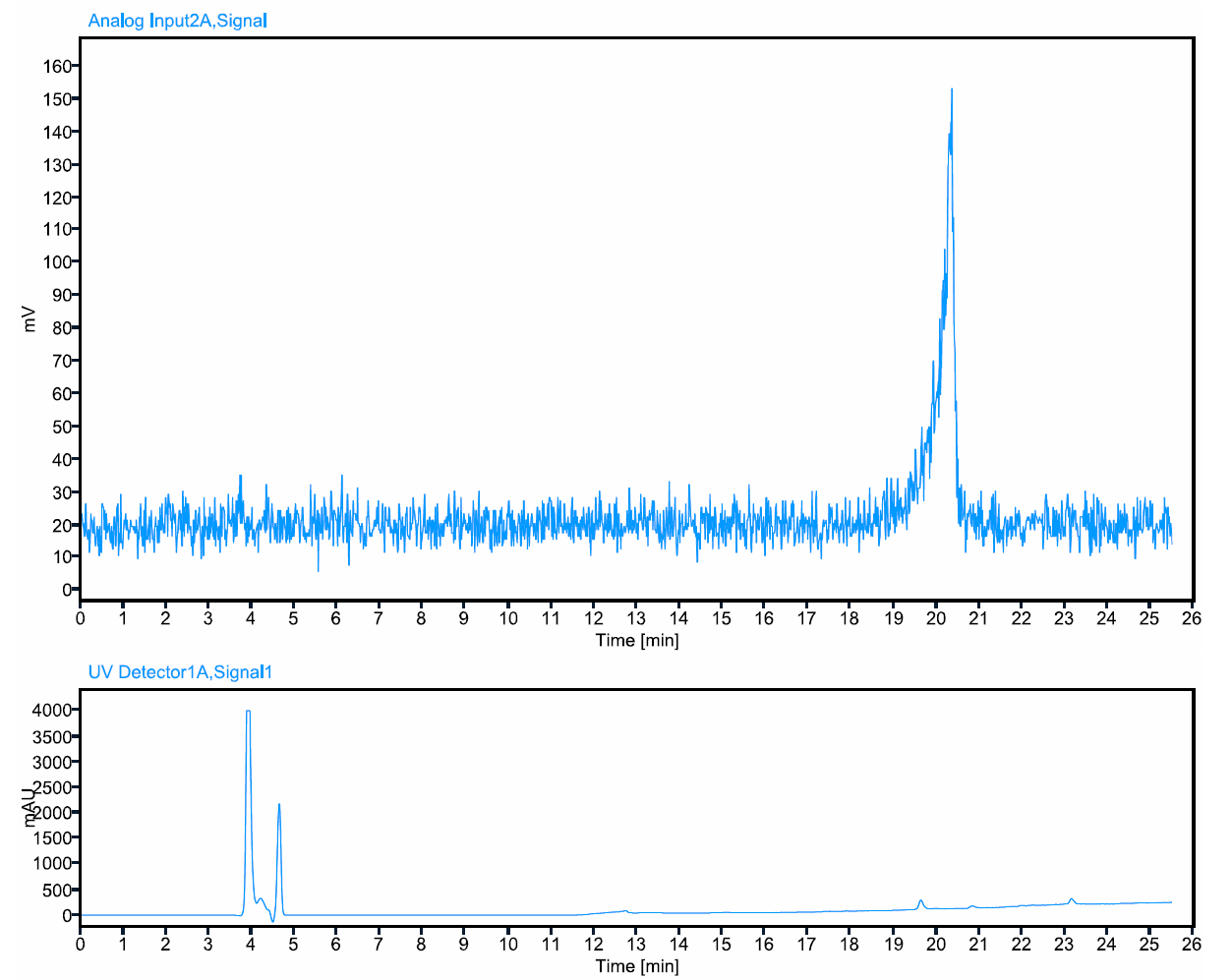


**a.**

**b.**

**c.**

**d.**

**e.**

***Table S3***. Radiolabeling efficiency of [^89^Zr]Zr-Compd **17**

|  | | **Radiolabeling efficiency (%) [^89^Zr]Zr-Compd 17** | |
| --- | --- | --- | --- |
| **Time** | | **RT** | **37°C** |
| **pH 7.0** | 15 min | 93 | 100 |
|  | 30 min | 100 | 100 |
|  | 60 min | 100 | 100 |

Radiolabeling efficiency of compound **17** with Zirconium-89 (pH 7.0, RT and 37 °C), measured in duplicate using iTLC eluted with a mobile phase consisting of 50 mM EDTA.
